# A refined low-dose murine model of *Mycobacterium ulcerans* infection to assess integrated immune networks in Buruli ulcer pathogenesis

**DOI:** 10.1128/mbio.01931-25

**Published:** 2025-08-18

**Authors:** Stephen Muhi, Isabelle J. H. Foo, Lukasz Kedzierski, Jessica L. Porter, Hayley A. McQuilten, Brian Howden, Katherine Kedzierska, Andrew H. Buultjens, Brendon Y. Chua, Timothy P. Stinear

**Affiliations:** 1 Department of Microbiology and Immunology, Doherty Institute for Infection and Immunity, The University of Melbourne 2281, Melbourne, Victoria, Australia; 2 Victorian Infectious Diseases Service, Royal Melbourne Hospital 90134, Parkville, Victoria, Australia; 3 WHO Collaborating Centre for Mycobacterium ulcerans. Victorian Infectious Disease Reference Laboratory (VIDRL), Doherty Institute for Infection and Immunity 534133 , Melbourne, Victoria, Australia; Universite de Geneve, Geneva, Switzerland

**Keywords:** *Mycobacterium ulcerans*, Buruli ulcer, neglected tropical skin diseases, vaccines, immunity, host response, animal models, laboratory animals

## Abstract

*Mycobacterium ulcerans,* the causative agent of Buruli ulcer (BU), has a low infectious dose (<10 colony-forming units [CFU]). BU animal infection models typically use challenge doses orders of magnitude higher than natural infection. These doses are unrealistic for vaccine trials and studies of immunity. Here, we address this issue and describe a murine tail infection model in two genetically distinct mouse strains (BALB/c and C57BL/6) using quality-controlled *M. ulcerans* challenge doses (10–20 CFU and 100 CFU). Over 24 weeks, we measured >70 clinical, immunological, and microbiological parameters. Principal findings included a 100% infection rate even at the lowest inoculum, but with a dose-dependent delay in lesion onset and disease progression for both mouse strains. Bacterial growth kinetics were similar between mouse strains, but there was a difference in immune profiles between mouse strains and between “low” (10 CFU) versus “high” (100 CFU) bacterial challenges. C57BL/6 mice exhibited more robust systemic cellular responses and more rapid lesion onset compared to BALB/c mice. Activated CD8^+^ T cells and dendritic cells were dominant in responses to low-dose infection in C57BL/6 mice. Murine low-dose *M. ulcerans* infection models provide confidence for future human Buruli ulcer challenge trials and will inform the development of effective vaccines and therapeutics.

**IMPORTANCE:**

Buruli ulcer (BU) is an infection of subcutaneous tissue caused by *Mycobacterium ulcerans*. This bacterial infection affects the lives of thousands of people each year across West and Central Africa and Australia. Recent research showed that as few as 2–3 *M. ulcerans* are sufficient to cause infection. Unfortunately, earlier laboratory studies have used unrealistically high bacterial doses to test new vaccines or to understand host responses, compromising subsequent conclusions. This research is significant because it takes a fresh approach to develop an experimental animal infection model for BU that uses a carefully calibrated and realistic infectious dose. The findings from assessing this new infection model are the foundation for an ambitious new program to develop a controlled human infection model for *M. ulcerans*, a platform for developing new therapies to prevent and treat BU.

## INTRODUCTION

*Mycobacterium ulcerans* is a slow-growing environmental bacterium that causes the neglected tropical skin disease Buruli ulcer (BU) (1). The distinctive pathology of BU is driven by the *M. ulcerans* toxin mycolactone, a polyketide-derived small molecule with potent immunosuppressive and cytotoxic properties (2). Observations by noted Australian microbiologist Frank Fenner recorded that 5–10 *M*. *ulcerans* bacilli inoculated into a mouse footpad were sufficient to cause infection, with a long incubation period, up to 5 months (3). Contemporary research supports these early observations, with an estimated mean incubation period in humans of 4.5 months and an ID_50_ (dose to infect 50%) of just 2–3 CFU in BALB/c mice (4–6). This low infectious dose is like other pathogenic mycobacteria, including *M. leprae* (the causative agent of leprosy) (7) and *M. tuberculosis* (8). However, *M. ulcerans* vaccine experiments in mice have typically employed quite high bacterial challenge doses, in the range of 10^4^–10^6^ colony-forming units (CFU) (9). In Australia, mosquitoes are the vector of *M. ulcerans*, with a relatively low burden of the pathogen associated with the insects, as established by qPCR (294 genomic equivalents [range, 11–4,200]) (10). Thus, findings from prior vaccine- and immunological-related investigations in mice may have confounded the protective potential of various experimental vaccines by overwhelming the host and inducing potentially irrelevant immune responses. Here, we sought to define in depth host-integrated immune responses after exposure to low *M. ulcerans* doses across 66 immune parameters, four different time points, and two mouse strains.

A major impediment to using low *M. ulcerans* challenge doses has been the technical issue of producing a cell bank with a reliable and reproducible concentration of viable bacteria. *M. ulcerans* is notoriously difficult to handle in the laboratory, as the cells are very hydrophobic and tend to clump (11, 12), making preparation of homogeneous cell suspensions difficult. In a previous study, we addressed this bottleneck and described a process to produce a quality-controlled cell bank of low-dose *M. ulcerans* JKD8049 (13)*,* for use in future controlled human infection models (14). We showed that this inoculum successfully established infection in 20 BALB/c mice, with a 100% attack rate achieved using a dose of only 20 CFU (15). The overarching goal of the study presented here was to define this quality-controlled cell bank of *M. ulcerans* JKD8049 by dissecting host responses to inform subsequent human challenge trials. The specific aims of our study were to understand the clinical and immunological responses to (i) a narrow range of low *M. ulcerans* challenge doses in (ii) genetically diverse mouse strains (BALB/c and C57BL/6 mice) that are known to have distinct immunological responses to intracellular infection (16). Pathogen responses are particularly important to examine in BU, as *M. ulcerans* exhibits both intracellular and extracellular phases of infection (17). Our study was also designed to inform initial dose-finding trials in humans, as the “attack rate” using a very low inoculum (≤20 CFU) was hypothesized to be lower in mice with a Th1-biased response to infection (i.e., C57BL/6 mice), particularly in controlling early (intramacrophage) infection, when mycolactone concentration is low (18).

## MATERIALS AND METHODS

### Inoculum preparation

*M. ulcerans* JKD8049, first isolated from a middle-aged male in Point Lonsdale, Victoria, Australia, was prepared and characterized as described previously (13). In brief, *M. ulcerans* JKD8049 was cultured in Sauton medium (SM) prepared as described previously (13), without the addition of surfactant (e.g., polysorbate/Tween) to minimize chemical modification of the mycobacteria. Animal-free and non-genetically modified vegetable peptone broth (Veggietones VG0101, Oxoid, Basingstoke, UK), 10% (vol/vol), was added as a growth supplement (“SMVT”). A mechanically filtered suspension of this culture was then cryopreserved at −80°C in 20% (vol/vol) glycerol. After thawing on ice, the vials were briefly vortexed prior to use. To confirm the final dose injected into mice, a 20 µL volume of a 10-fold dilution series of the suspension was spotted onto Middlebrook 7H10 agar (BD, Sparks, MD, USA) enriched with 10% oleic acid, albumin, dextrose, and catalase (OADC; HiMedia, Mumbai, India). The sample was then drawn into a 1 mL low dead-space (LDS) syringe (B-Braun Omnifix-F, Melsungen, Germany); the sample volume was determined based on the CFU count from other vials produced in the same batch, aiming for two doses: 10–20 CFU/mouse and 100 CFU/mouse, in a maximum final volume of 50 µL/dose. A separate 1 mL syringe, containing phosphate-buffered saline (PBS) as diluent, was connected to the syringe containing the sample using a Luer lock connector (B-Braun Fluid Dispensing Connector, Melsungen, Germany). Mice receiving a “high dose” of *M. ulcerans* JKD8049 were administered a mean of 99 CFU in 50 µL for each mouse challenge dose (95% confidence interval [CI] 77–124 CFU); mice receiving a “low dose” each received a mean of 11.2 CFU (95% CI 9.5–12.5 CFU) in 50 µL.

### Mice challenge procedure

Eight-week-old female BALB/c and C57BL/6 mice were infected with *M. ulcerans* JKD8049, with 5 mice of each strain challenged with “high-dose” or “low-dose” *M. ulcerans*. All mice were lightly anesthetized with isoflurane and infected subcutaneously in the dorsal surface of the proximal tail, with the needle advanced approximately 3 mm proximally below the skin directly between the lateral tail veins in a 50 µL volume, as previously described (15). Five control mice of each strain were injected with an identical volume of media only; SMVT was prepared as described previously (13) in 20% glycerol (vol/vol).

### Harvesting of tissues and collection of blood

Spleen, inguinal draining lymph nodes (DLN), tail sections, and blood were collected at predefined time points after challenge, with 5 mice of each strain culled at each timepoint (4, 6, 10, 14, and 28 weeks after challenge, or at the onset of ulceration). Spleens and DLNs were collected in 3 mL Roswell Park Memorial Institute (RPMI; Life Technologies) medium containing 2% fetal calf serum (FCS) (vol/vol) and passed through 70 µm cell sieves (Miltenyi Biotech) to obtain single-cell suspensions. Splenic-derived cell suspensions were further treated with 0.15 M NH_4_Cl and 17 mM Tris-HCl at pH 7.2 for 5 minutes at 37°C to lyse red blood cells and washed with medium prior to analysis. Blood was collected from mice immediately post-mortem from a terminal cardiac bleed. Blood was then placed on ice for 4–6 hours, after which clots were removed manually. The samples were then centrifuged at 2,000 rpm for 5 minutes at 4°C, and the supernatant was collected for cytokine and chemokine analysis and antibody responses. Tail tissue was prepared for analysis as described previously (4). Briefly, tail sections without lesions were harvested by dissecting the proximal third of the tail, and ulcerated tails were dissected with a small margin on either side of the lesion. For homogenization, tail sections were placed in 600–1000 µL of PBS and processed by bead beating using ~0.5 g 2 mm glass beads in a high-speed tissue-disruptor (Precellys 24, Bertin Technologies, France) at 6,500 rpm, with 4 rounds of 2 × 30 second pulses (with tubes placed on ice for 5 minutes between each round). Homogenized tissue samples were stored at −80°C.

### Immunophenotypic staining

Cells were stained with combinations of fluorochrome-conjugated antibodies (clone; catalog number; dilution). BD Biosciences: anti-CD8α-PerCyP Cy5.5 (53-67; 551162; 1:200), anti-CD4-BV650 (RM4-5; 563747; 1:200), anti-CD25-PECF594 (PC61; 562694; 1:200), anti-CD45-PerCP (30-F11; 557235; 1:200), anti-CD38-BV711 (90; 740697; 1:200), anti-CD138-PE (281-2; 553714; 1:400), anti-CD11b-BV605 (M1/70; 563015; 1:600), anti-SigLecF-PECF594 (E50-2440; 562757; 1:1200), anti-Ly6G-PECy7 (1A8; 560601; 1:1200), anti-CD19-BV605 (1D3, 563148, 1:200), anti-CD27-PE (LG.3A10, 558754, 1:200), anti-CD49b-APC (DX5, 560628, 1:100), anti-I-A/I-E-AF488 (M5/114, 562352, 1:200), anti-iNOS-FITC (6/iNOS/NOS Type II, 610331, 1:100), anti-KLRG1-APC (2F1, 561620, 1:200), anti-NK1.1-AF700 (PK136, 560515, 1:100), and anti-TCRγδ-BV421 (GL3, 562892, 1:200).

BioLegend: anti-CD62L-BV570 (MEL-14; 104433; 1:200), anti-CD279-BV785 (29F.1A12; 135225; 1:200), anti-CD69-PECy7 (H1.2F3; 104512; 1:200), anti-GL7-PerCyPCy5.5 (GL7; 144610; 1:200), anti-IgD-APC Cy7 (11-26c.2a; 405716; 1:200), anti-CD3-BV785 (145-2C11; 100355; 1:200), anti-F4/80-APC (BM8; 123116; 1:150), anti-CD45R-BV650 (RA3-6B2, 103241, 1:200), anti- CD107a-BV421 (1D4B, 121617, 1:100), anti-CD11c-BV650 (N418, 117339, 1:200), anti-CD44-APC Cy7 (IM7, 103028, 1:200), anti-CD64-BV421 (X54-5/7.1, 139309, 1:100), and anti-Ly6C-BV570 (HK1.4, 128030, 1:200).

eBiosciences: anti- I-A/I-E-APC-eFlour780 (M5/114.15.2; 47-5321-82; 1:200), anti-Arg1-AF700 (A1exF5, 56-3697-82, 1:100), anti-CD206-PE (MR6F3, 12-2061-82, 1:100), and anti-CD3-AF700 (eBio500A2, 56-0033-82, 1:200).

Cell viability was determined by staining with Live/Dead-Aqua 525 (L34966A, ThermoFisher, 1:800). Antibody staining was performed at 4°C in the dark. Cells were fixed with 1% paraformaldehyde before analysis by flow cytometry. Samples were subsequently acquired on a BD LSR Fortessa (BD Biosciences) flow cytometer and data analyzed by FlowJo Software (Tree Star Inc., USA). Gating strategies for flow cytometry data are shown in Fig. S1.

### Establishing colony counts

A 300 µL volume of tail tissue homogenate was decontaminated with 300 µL of 2% NaOH (vol/vol) and incubated at room temperature for 15 minutes. The preparation was then neutralized with 50 µL of 10% orthophosphoric acid (vol/vol). 300 µL of this mixture was then diluted in 300 µL PBS, and CFUs were determined by spot plating as described previously (4). In brief, 6 × 5 µL volumes of serial 10-fold dilutions (10^−1^ to 10^−5^) of tissue preparation were spotted onto 7H10/OADC agar plates with a 5 × 5 grid (10 cm × 10 cm). A second plate (i.e., technical replicate) was also spotted in case of contamination. The spots were allowed to dry, loosely wrapped in a plastic bag, and incubated for 12 weeks before final colony count. Plates were checked every 2 weeks.

### Histopathology

In groups of mice without any visible lesion, two of five mice tails were dissected for histology using the entire proximal third of the tail. In mice with a visible lesion, the segment of involved tail tissue was divided into two equal sections. Samples of excised tissue were fixed in neutral buffered formalin, decalcified in 10% formic acid for 48 hours, and embedded in paraffin for histopathological examination. Tissue sections were then stained with hematoxylin and eosin (HE) and Ziehl-Neelsen (ZN) for light microscopy. Digital images were captured using a Pannoramic SCAN II (3D Histech), using a Plan-Apochromat 20×/NA 0.8 objective (Carl Zeiss Microscopy) and a Grasshopper 3 CCD monochrome camera (Point Grey).

### DNA extraction and PCR

DNA was extracted from homogenized tail tissue using a DNeasy PowerSoil kit (Qiagen). Control mice were included to monitor potential PCR contamination in addition to no-template negative PCR controls. IS*2404* quantitative PCR (qPCR) was performed using technical duplicates as described previously (19). IS*2404* cycle threshold values were converted to genomic equivalents as described previously (4). In brief, cycle thresholds were converted using a reference standard curve calculated using dilutions of genomic DNA from *M. ulcerans* JKD8049 (4).

### Serum cytokines and chemokines analysis

Cytokine and chemokine levels in mice sera were analyzed using the Mouse LEGENDplex MU Cytokine Release Syndrome Panel (13-plex) kit (Biolegend) according to the manufacturer’s instructions.

### Antibody responses

*M. ulcerans* whole-cell lysate (WCL) was prepared using *M. ulcerans* JKD8049 cultured on solid agar for >12 weeks. Culture material was scraped into 2 mL vials containing ~0.5 g 2 mm glass beads and 1 mL of sterile PBS and homogenized in a high-speed tissue-disruptor. Homogenized lysate was then centrifuged at 4,000 rpm for 5 minutes, and the supernatant was transferred into a separate container. The final protein concentration (0.4 mg/mL) was determined using a Qubit Protein Assay (Invitrogen), as per the manufacturer’s instructions for use.

Enzyme-linked immunosorbent assays (ELISAs) were performed on Nunc Immuno MaxiSorp PS flat-bottom 96-well plates (Thermo Scientific). Each well was coated with 50 µL of PBS containing 5 µg/mL of WCL and incubated overnight at 4°C. The following day, WCL was discarded, and 100 µL of PBS containing wt/vol 10% bovine serum albumin (BSA_10_PBS) was added to each well. This was incubated for 1 hour at room temperature (RT). The BSA_10_PBS was then discarded, and the plate washed four times with PBS containing vol/vol 0.05% Tween (PBST) and once with PBS. Plasma was added to the wells in a log_0.5_ dilution series in PBS containing vol/vol 0.05% Tween and wt/vol 5% PBS (BSA_5_PBST), and incubated for 2 hours at RT. Following the discarding of well contents, each plate was washed as described. For immunoglobulin detection, 50 µL of polyclonal rabbit anti-mouse Ig-HRP (Dako) was added to each well at a 1:2,000 dilution in BSA_5_PBST and incubated for 1 hour at RT. The content of each plate was then discarded and washed seven times with PBST and once with PBS, followed by the addition of 100 µL of tetramethylbenzidine (TMB; Sigma) and allowed to develop for 10 minutes before 100 µL of 1 M orthophosphoric acid was added. Plates were then read immediately at an absorbance wavelength of 450 nm on a Multiskan MS multiplate reader (Labsystems). Endpoint titers were determined by interpolation from a sigmoidal curve fit (all R-squared values > 0.95; GraphPad Prism 10) and expressed as the reciprocal of the highest dilution of plasma required to produce an absorbance value of 0.8. Intra-experimental measurements were normalized using a positive control plasma sample included on each plate.

### Statistical analysis

Statistical analysis was performed using GraphPad PRISM (version 10.1.1). Continuous variables are reported as mean/standard deviation or 95% confidence interval (of the mean), as relevant. Comparisons between groups were performed using Student’s t-test (for difference between means) and illustrated using Kaplan-Meier curves using the log-rank test, with statistical significance if *P* < 0.05. Univariate Mann-Whitney U tests were performed in Python using SciPy (version 1.9.1) to compare individual immune features between the low- and high-dose groups. To adjust for false discovery due to multiple comparisons, from each group of highly correlated variables (defined as a correlation coefficient >0.8), only one representative variable was retained to minimize redundancy. The Benjamini–Hochberg procedure was then applied, with statistical significance defined as *P* < 0.05.

Locally Weighted Scatterplot Smoothing (LOWESS) regression was conducted in Python (v3.10.6) using pandas (v1.4.2) and numpy (v1.23.3), with visualizations created in matplotlib (v3.7.1). Linear models of log-transformed variables were constructed in R (v4.2.1) and plotted with ggplot2 (v3.3.5). Heatmaps were generated using seaborn (v0.12.2), where values for each immune parameter were normalized across all timepoints and groups (control, low-, and high-dose challenge).

Random Forest classifiers were applied to evaluate the ability of immune parameters to distinguish between low- and high-dose challenge groups using sklearn (v1.1.2). The data were divided into 10 stratified folds, and feature importances were averaged across folds. Classifier performance was assessed by calculating the area under the receiver operating characteristic curve (AUC), with results aggregated across all folds. To establish a null distribution, the analysis was repeated 100 times with randomized class labels and AUC scores from these runs were compared to the actual data using density plots.

Pearson correlation coefficients were calculated in R version 4.4.1 using the psych (v2.4.6.26) package function corr.test, with *P*-values representing the Benjamini-Hochberg false discovery rate adjustment for multiple t-tests. Pairwise correlation plots were made using ggplot2 version 3.5. Correlation matrices were made using corrplot (v0.95).

## RESULTS

### Low challenge doses of *M. ulcerans* JKD8049 lead to tail lesion formation and progression of clinical disease in both C57BL/6 and BALB/c mice

**Fig 1:**
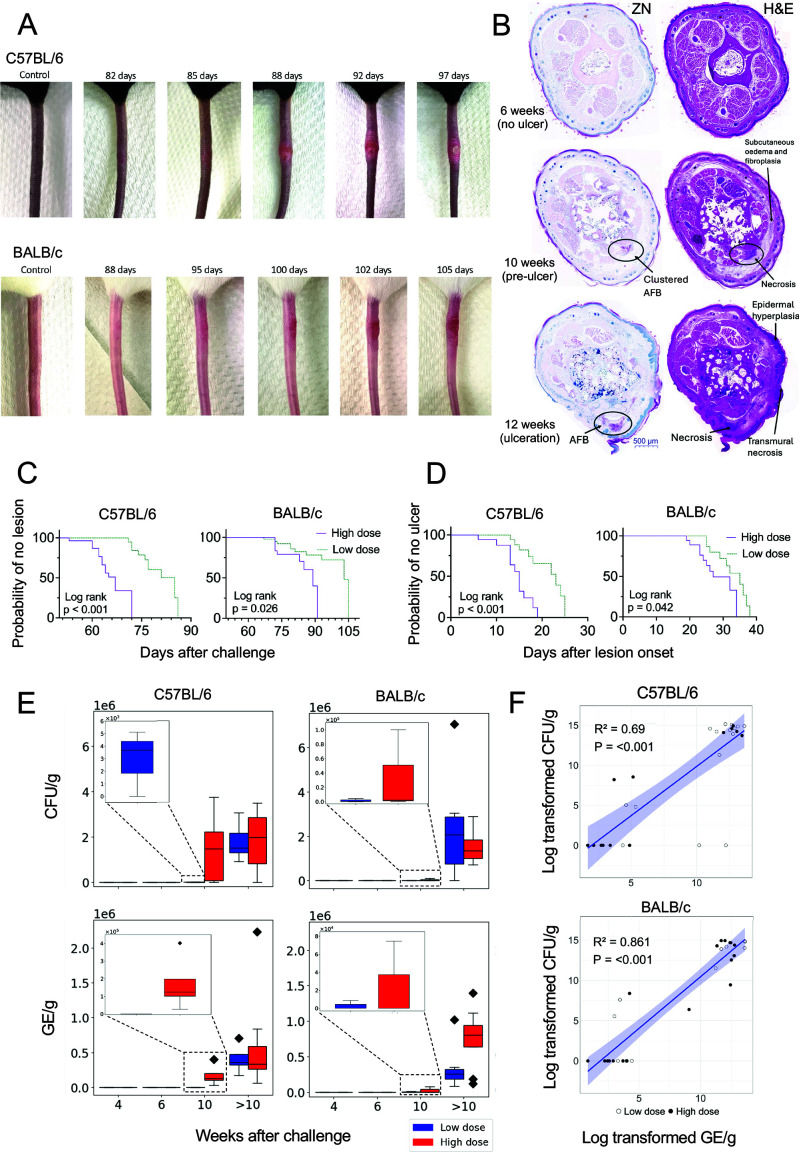
Clinical and microbiological features of low-dose infection over time. (**A**) Typical progression of tail lesions in female C57BL/6 (top row) and BALB/c (bottom row) mice challenged subcutaneously with ~100 CFU of *M. ulcerans* JKD8049. (**B**) Histopathological features of C57BL6 mouse tail imaged on day 42, 70, and 84 following challenge with ~100 CFU of *M. ulcerans* JKD8049. Tail sections were stained with Ziehl-Neelsen (ZN) acid-fast stain and hematoxylin and eosin (H&E) stain; representative images are shown. Bar represents 500 µm length at magnification of 2× The circled area shows the presence of acid-fast bacilli (AFB), and the arrows point to areas of histopathology. (**C**) Differences in the time to lesion onset in BALB/c and C57BL/6 mice depending on the challenge “dose” received (~10 CFU challenge dose: *n* = 10 per group; ~100 CFU challenge dose: C57BL6 *n* = 15, BALB/c *n* = 10). (**D**) Time from lesion onset to ulceration in these mice (~10 CFU challenge dose: *n* = 10 per group; ~100 CFU challenge dose: C57BL6 *n* = 12, BALB/c *n* = 10). Comparisons between groups were performed using the log-rank test. (**E**) Bacterial growth over time in CFU/g and genomic equivalents (GE)/g. (**F**) Relationship between the log-transformed CFU/g and GE/g in both mouse groups (C57BL/6 top, BALB/c bottom); comparisons between groups were performed using linear models constructed in R (v4.2.1) on log-transformed variables (selected using the bestNormalize package). Model fit was evaluated using R², while the significance of association was determined using *P*-values from t-tests on the model coefficients.

To compare the pathology and immunology of different *M. ulcerans* dosing strategies, we challenged C57BL/6 and BALB/c mice with a low and high dose of *M. ulcerans*, corresponding to an average 11.2 CFU (95% CI 9.5–12.5 CFU) and 99 CFU (95% CI 77–124 CFU) (Fig. S2A), respectively. We then followed responses to infection at 4, 6, and 10 weeks following challenge, and at the onset of tail ulceration. Negative control mice were challenged with culture media only. In both C57BL/6 and BALB/c mice receiving either dose, typical BU lesions were observed in all mice, namely, lesions began as a small patch of erythema with hair loss that progressed to enlarging induration and eventual ulceration (Fig. 1A).

Histopathology of tail sections for C57BL/6 mice at 10 weeks post-low-dose challenge showed evidence of pre-ulcerative disease, with mild focal subcutaneous edema and scanty lymphocytic infiltration. No acid-fast bacilli (AFB) were detected by Ziehl-Neelsen (ZN) staining at this time point (Tables S1 and S2). These inflammatory features were not as apparent for similarly challenged BALB/c mice (Tables S3 and S4). In C57BL/6 mice that received the high dose, the pathology in tail sections was prominent at earlier time points and typified by moderate multifocal to diffuse subcutaneous edema. Two of five of these mice at the 10 week time point showed mononuclear cell infiltration, while three mice had a mixed inflammatory infiltrate associated with vasculopathy and skeletal myofiber necrosis (Fig. 1B). One C57BL/6 mouse at this time point also had intracellular AFB visible within macrophage cytoplasm. By comparison, in the low-dose C57BL/6 mice examined 10 weeks after infection, the two mice examined histologically both had mild subcutaneous edema and scanty lymphocytes, with no AFB visible. At later time points (>10 weeks), there was visible ulceration in both dose groups and mouse strains. Common histological features in these mice included subcutaneous edema with a mixed infiltrate, vasculopathy, myonecrosis, and abundant AFB. Of note, BALB/c mice culled late in the low-dose challenge group also showed chronic, active inflammation that was not observed in any mice given a high dose. Moreover, in all mice with visible lesions, 3 of 25 mice in the high-dose cohorts had contiguous AFB detected in the underlying bone marrow. This phenomenon was not detected in any of the mice with visible lesions (*n* = 19) challenged with the low dose of *M. ulcerans* (Fig S2B; Tables S1 and S3).

### Low challenge doses of *M. ulcerans* result in a longer incubation period and slower progression of clinical disease in both BALB/c and C57BL/6 mice

There was a clear dose-response relationship in both mouse strains. Low-dose mice, irrespective of strain, developed lesions later than those receiving the high dose (Fig. 1C). Mice receiving a low dose of *M. ulcerans* also had a slower progression of clinical disease (the time from lesion onset to ulceration), regardless of mouse strain (Fig. 1D). In addition, despite infecting with identical bacterial doses, disease onset and disease progression were significantly slower in BALB/c mice relative to C57BL/6 mice (Fig S2C and D).

In C57BL/6 mice, the mean time-to-lesion-onset (i.e., incubation period) was 64 days for the high dose compared to ~77 days following a low dose (delay in symptom onset was 13 days) (Table 1). Similarly, visible lesions were also observed in all infected BALB/c mice, with a mean time-to-lesion onset of ~79 days in those that received a high dose compared to ~84 days for the low dose (delay in symptom onset ~5 days).

**TABLE 1:** Clinical parameters of mice allowed to progress to clinical disease, depending on mouse type and dose of *M. ulcerans* JKD8049^*a*^

Mouse type	Dose group	Attack rate (%)	Mean incubation (days) (95% CI)	Mean time from lesion onset to ulcer (days) (95% CI)	Delay in incubation period (days)	Delay in time from lesion onset to ulceration (days)
C57BL/6	High	15/15 (100)	64.1 (53.0–72.0)	13.4 (11.7–16.1)		
C57BL/6	Low	10/10 (100)	77.1 (71.0–86.0)	19.6 (16.2–23.0)	13.0	5.7
BALB/c	High	10/10 (100)	79.1 (72.0–89.0)	25.3 (21.9–28.7)		
BALB/c	Low	10/10 (100)	83.8 (74.6–93.0)	31.4 (27.8–35.0)	4.7	6.1

^*a*^ Empty cells indicate the 'delay in incubation period' and ' delay in time from lesion onset to ulceration' are between the high and low dose challenge models for each mouse strain, so this is only required twice in the table.

Upon appearance of visible lesions, the mean time from lesion onset progressing to ulceration in C57BL/6 mice was ~14 days following a high dose and ~20 days in those that received a low dose (delay in ulceration ~6 days) (Table 1). In comparison, the mean time from lesion onset to ulceration in BALB/c mice was ~25 days in high-dose mice and ~32 days for low dose (delay in ulceration ~6 days). These results demonstrate dose-dependent disease outcomes with a high dose exhibiting more rapid appearance of lesions and development of ulcers in both mouse strains. There was also a faster rate of symptom progression in C57BL/6 mice compared to BALB/c mice, irrespective of the dose.

Of C57BL/6 mice that developed ulceration, some weight gain was observed compared to uninfected controls, but this change was not statistically significant (Table S5). No weight change was observed for BALB/c mice (Table S6). All infected mice appeared otherwise healthy and did not appear to groom or attend to their lesions, and no secondary lesions were observed.

### Exponential *M. ulcerans* growth occurs in both BALB/c and C57BL/6 mice after both high and low challenge doses

To characterize bacterial growth dynamics during infection, IS*2404* qPCR and CFU counts were performed on mouse tail homogenates. *M. ulcerans* was detected by culture in tail tissue from high-dose C57BL/6 mice from 6 weeks post-inoculation (Table 2; Fig. 1E). Despite the absence of any visible lesion, two of three samples collected at 6 weeks resulted in bacterial colony growth (mean 136 CFU/g). By contrast, low-dose C57BL/6 mice were culture positive from 10 weeks, at which time two of three samples were positive in the absence of any lesion (mean 4.39 × 10^3^ CFU/g). Tail tissue from BALB/c mice was culture positive from 10 weeks, where all three tails tested from the high-dose challenge cohort were positive (mean 3.41 × 10^4^ CFU/g) compared to two of three tails in the low-dose challenge group (mean 2.47 × 10^3^ CFU/g). All mice culled >10 weeks after challenge had ulcerative disease and bacterial counts were high in these mice (mean 2 × 10^6^ CFU/g) irrespective of low- or high-dose inoculum. There were no differences in *M. ulcerans* growth kinetics between BALB/c and C57BL/6 mice (Fig S2E). The lower dose resulted in a later time to detection of viable bacilli in both mouse strains. Using *M. ulcerans* qPCR that is more sensitive and less resource-intensive than culture, we observed an exponential pattern of growth by plotting genomic equivalents (GE) per gram of tail tissue over time (Fig. 1E; Fig S2F). There was a significant positive correlation between CFU/g and GE/g in both mouse strains (Fig. 1F).

**TABLE 2:** *M. ulcerans* JKD8049 CFU count and genomic equivalents in mouse tail homogenate^*a*^^,^^*b*^

Mice and weeks after challenge	No. culture positive	No. with lesion	Mean CFU/g	SD	Mean GE/g	SD
C57BL/6, high dose						
4	0 of 3	0	N/A	N/A	83	16
6	2 of 3	0	136	23	128	69
10	4 of 5	5	1.88E + 06	1.54E + 06	1.70E + 05	1.43E + 05
>10	10 of 10	10	1.64E + 06	1.15E + 06	2.96E + 05	1.75E + 05
C57BL/6, low dose						
4	0 of 3	0	N/A	N/A	16	12
6	0 of 3	0	N/A	N/A	11	5
10	2 of 3	0	4.39E + 03	1.01E + 03	114	68
>10	7 of 10	10	2.21E + 06	1.00E + 06	5.90E + 05	6.29E + 05
BALB/c, high dose						
4	0 of 3	0	N/A	N/A	48	8
6	0 of 3	0	N/A	N/A	53	28
10	3 of 3	0	3.41E + 04	5.71E + 04	2.49E + 04	4.30E + 04
>10	10 of 10	10	2.31E + 06	2.23E + 06	9.24E + 05	5.17E + 05
BALB/c, low dose						
4	0 of 3	0	N/A	N/A	19	19
6	0 of 3	0	N/A	N/A	10	2
10	2 of 3	0	2.47E + 03	2.67E + 03	2.92E + 03	4.95E + 03
>10	10 of 10	10	2.20E + 06	2.03E + 06	3.08E + 05	2.63E + 05

^*a*^ CFU: colony forming units; SD: standard deviation; GE: genomic equivalents.

^*b*^ Not applicable (as none of the cultures were positive, there is not mean or standard deviation).

In C57BL/6 mice, the higher dose correlated with faster time-to-lesion onset (Fig. 1C) and more rapid progression of clinical disease (Fig. 1D). In BALB/c mice, although a similar pattern emerged, the resolution between high and low challenge doses was less apparent, suggesting that clinical outcomes are less readily distinguished between the two doses in BALB/c mice (Fig. 1C and D). In C57BL/6 mice, despite mice progressing faster to visible clinical disease following the high dose, there were similar or greater bacteria in mice at the time of ulceration that received the low dose (Fig S3A and B). This suggests that pathology at these clinical endpoints may be driven by non-bacterial processes in C57BL/6 mice, a pattern that was not as apparent in BALB/c mice.

### C57BL/6 mice demonstrate an early splenic response to low-dose infection, followed by a late response in draining lymph node tissue

**Fig 2:**
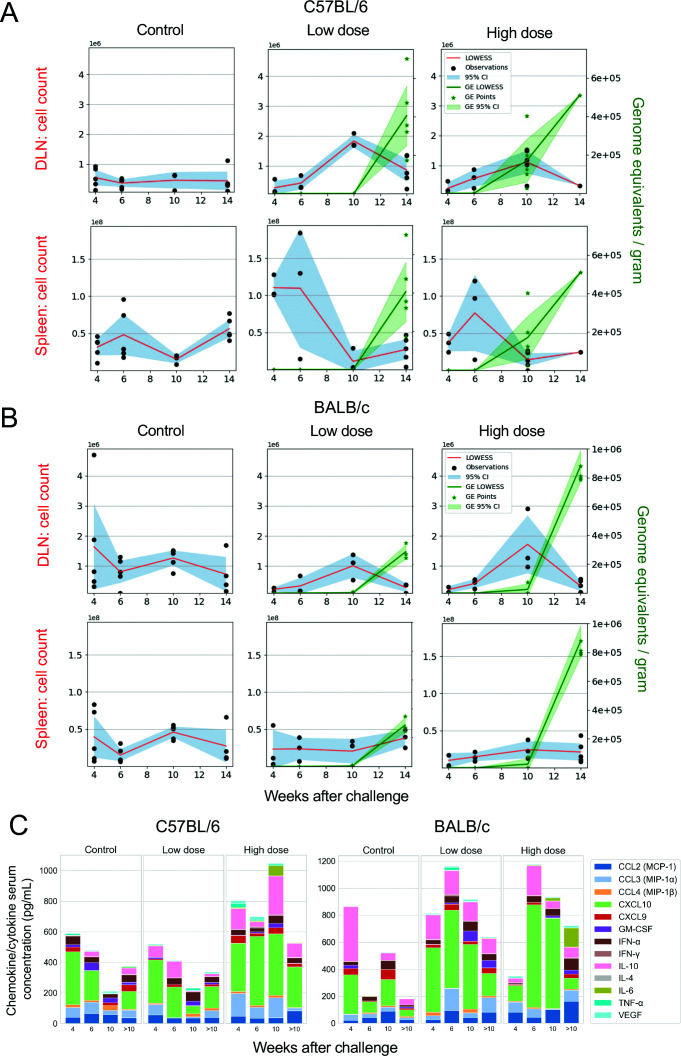
Cellular and cytokine/chemokine features over time. (**A**) Total cell counts observed in C57BL/6 and (**B**) BALB/c mice at 4, 6, and 10 weeks after challenge (as per protocol), in splenic tissue and draining lymph nodes following either media-only “sham” (control mice, left column) or infectious challenge. Mice that received a “low-dose” challenge are visualized in the center column, and mice that received a “high dose” of *M. ulcerans* are visualized in the right column. Images also include mice culled 14 weeks after challenge, at which time mice had reached the human end-point (ulceration). The blue color represents the 95% CI around the Locally Weighted Scatterplot Smoothing (LOWESS) line (in red), and the green color represents the genomic equivalents (GEs) LOWESS line and 95% CI (C57BL/6 mice controls: week (**W**) 4–6 *n* = 5 per group, W10 *n* = 3, W14 *n* = 5; C57BL/6 ~ 10 CFU dose: W4 *n* = 4, W6 – 14 *n* = 5 per group; C57BL/6 mice ~ 100 CFU dose: W4 – 10 *n* = 5 per group, W14 *n* = 1; BALB/c mice controls: W4 – 6 *n* = 5 per group, W10 – 14 *n* = 4 per group; BALB/c mice ~ 10 CFU dose: W4 – 10 *n* = 5 per group, W14 *n* = 3; BALB/c mice ~ 100 CFU dose: W4 *n* = 4, W6 – 14 *n* = 5 per group). (**C**) Serum cytokine and chemokine concentrations over time across a maximum of 140 days were assayed by LegendPlex; C57BL/6 mice controls: W4 – 6 *n* = 5 per group, W10 *n* = 3, W > 10 *n* = 12; C57BL/6 mice, ~10 CFU dose: W4 *n* = 4, W6 – 10 *n* = 5 per group, W > 10 n =10; ~100 CFU dose: W4 –10 *n* = 5 per group, W > 10 *n* = 10; BALB/c mice controls and both dosing groups: W4 – 10 *n* = 5 per group, W > 10 *n* = 10.

We next investigated immunological responses over time systemically, as captured in splenic tissue, and peripherally in the inguinal draining lymph nodes (DLNs) that are proximal to the site of challenge at the base of the tail. We first analyzed immune cell populations in the spleen and DLNs over the course of infection progression. Compared to uninfected C57BL/6 mice, increased immune cell numbers in the spleen were apparent 4–6 weeks following low-dose challenge, but decreased to baseline levels from 10 weeks onwards, concomitant with a rise in bacterial numbers (Fig. 2A). A similar peak in cell numbers was observed in those mice that received the high dose, albeit occurring later at 6 weeks, consistent with an earlier increase in bacterial burden at 10 weeks. By contrast, cell numbers in the DLNs did not peak until 10 weeks after challenge, most prominently in the low-dose group, and like observations in the spleen, DLN cell numbers subsided as bacterial concentrations increased. However, in BALB/c mice challenged with either dose, there were no discernible changes in splenic and DLN cell numbers over the course of the following 14 weeks, despite increases in the bacterial burden at the humane endpoint, with the exception of the DLNs from those mice that received the higher dose, which saw an increase in cell numbers at 10 weeks post-challenge (Fig. 2B).

### Cytokine/chemokine responses are dose-dependent and more prominent in C57BL/6 mice

An analysis of 13 serum cytokines and chemokines in C57BL/6 mice demonstrated that only IL-6 was greater in high dose mice than low dose (*P* = 0.005) (Fig. 2C; Fig S4A; Table S7). In BALB/c mice, the pattern of cytokine/chemokine responses was similar between the doses, peaking 6 weeks following infection, then subsiding (Fig. 2C). IFN-γ was significantly greater in the high-dose mice compared to the low dose early in the course of infection (*P* = 0.045). IL-10 was significantly greater in the high-dose mice compared to low-dose and control mice 10 weeks after challenge (*P* = 0.045, *P* = 0.044, respectively) (Fig. 2C; Fig S4B; Table S7). Given the role of IL-10 in regulating immune responses in general and the elevated IL-10 responses with inversely low IFN-γ expression in humans with BU, the levels of these cytokines may at least partially explain the reduced inflammatory response observed in BALB/c mice (20).

### Differential systemic versus local innate and adaptive cellular immune responses between mouse strains and between doses

**Fig 3:**
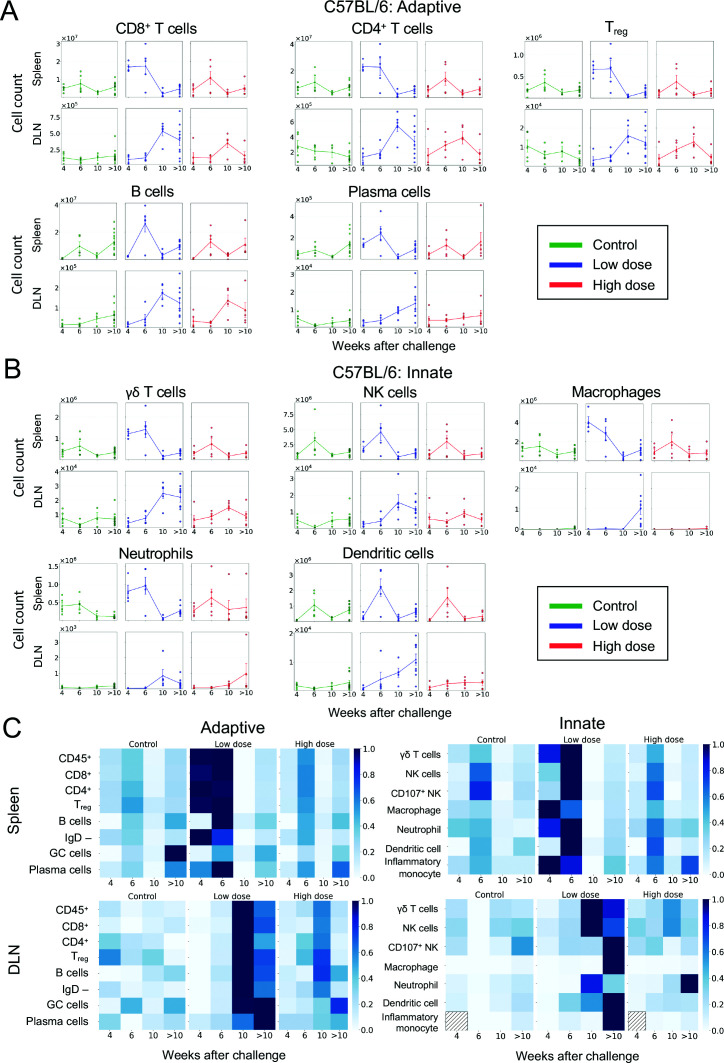
Total cell counts of adaptive and innate immune features over time in C57BL/6 mice following low-dose *M. ulcerans* infection. (**A**) Adaptive and (**B**) innate immune features over a maximum of 15 weeks using absolute numbers of effector CD8+ T cells (CD8+ CD62Llo CD44hi), effector CD4+ T cells (CD4+ CD62Llo CD44hi), Treg cells (CD4+ CD25+ CD62L+), B cells (CD19+ B220+), plasma cells (antibody secreting cells) (IgD-B220lo CD138+), γδ T cells (TCRγδ CD3+), NK cells (CD49b+ NK1.1+), macrophages (CD64+ F4/80+), neutrophils (CD11b+ Ly6G+), and dendritic cells (CD11c+MHCII+). The error bar represents the standard error of the mean (SEM). Statistically significant differences between groups are reported in Table S7 using Mann-Whitney U test. Gating strategy is shown in Fig. S1A–C. Source data are provided as a Source Data file. (**C**) Heatmap of normalized values demonstrates increased numbers of cells in splenic tissue at earlier time points (4–6 weeks after infection) followed by increased numbers of cells in draining lymph node (DLN) at later time points (10 and >10 weeks) after infection (C57BL/6 mice controls: week (W) 4–6 n = 5 per group, W10 n = 3, W > 10 n = 12; ~10 CFU dose: W4 n = 4, W6–10 n = 5 per group, W > 10 n = 9; ~100 CFU dose: W4–10 n = 5 per group, W > 10 n = 10).

Innate and adaptive immune cell populations over time were further analyzed to understand immune features influencing differences between dosing groups. In C57BL/6 mice, there were several adaptive immunity cell populations that were significantly different in number between dosing groups and controls (Fig.3A). In splenic tissue of C57BL/6 mice after low-dose challenge at 4 weeks, when compared to control mice, there were increased numbers of CD8^+^ and CD4^+^ T cells, T_reg_ cells, B cells, and plasma cells (all *P* = 0.016) (Table S7). In splenic tissue of high-dose C57BL/6 mice, these early differences were not apparent, and in some cases, numbers of these adaptive immune cells were significantly less than those observed in low-dose mice, particularly CD8^+^ T cells, CD4^+^ T cells, T_reg_ cells, B cells (all *P* = 0.016), and plasma cells (*P* = 0.032) at 4 weeks (Table S7).

There were also significant differences in innate immune cell numbers in C57BL/6 mice. In splenic tissue, 4–6 weeks after low-dose challenge, compared to control mice, there were increased levels of γδ T cells, neutrophils, macrophages (all *P* = 0.016), and dendritic cells (*P* = 0.032) (Fig. 3B; Table S7). Following high-dose challenge, differences in innate splenic immune responses were not as apparent and, in some cases, were again significantly below values observed in the low-dose group, including γδ T cells, macrophages (both *P* = 0.016), and dendritic cells (*P* = 0.032) at 4 weeks (Table S7).

In the interval 6–10 weeks after challenge, there were no significant differences between the two C57BL/6 dosing groups. In mice that had reached the humane end point (i.e., those culled >10 weeks after challenge), there were significantly more macrophages (*P* =< 0.001) and dendritic cells (*P* = 0.012) in DLNs of mice challenged with a low dose compared to the high dose, and numerous adaptive and innate immune features were significantly greater in the low-dose model compared to controls (Table S7).

With multiple immune features compared over various time points, adjustment for repeated measures was performed to understand which comparisons were most unlikely to be detected by chance alone, including markers of activation. This false discovery adjustment highlighted the significance of adaptive cells, particularly CD8^+^ cells, in C57BL/6 responses to *M. ulcerans* low-dose infection. In C57BL/6 mice with ulcerative disease, activated CD38^+^CD8^+^ cells were significantly more numerous in DLNs of low-dose mice compared to both controls and high-dose mice (Table S8).

When immune cellular values were normalized and compared over time, an early splenic increase in cell numbers, followed by later increases in DLNs, was most apparent in the C57BL/6 low-dose model (Fig. 3C). The blunted cellular immune responses observed in the high-dose-infected mice might be explained by presumed higher mycolactone concentrations associated with the increased bacterial burden.

### BALB/c cellular responses to infection are not dose-dependent within the dose range tested

**Fig 4:**
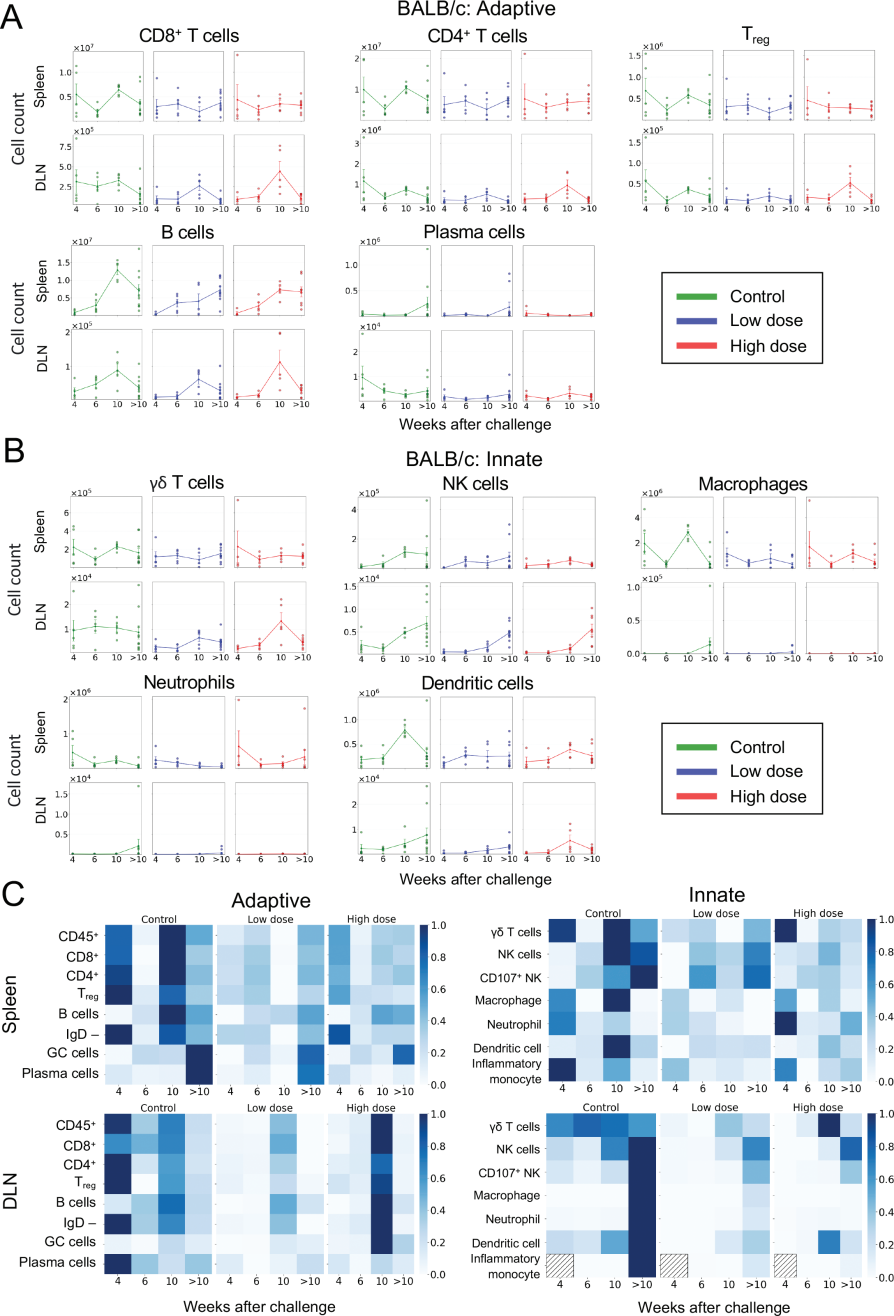
Total cell counts of adaptive and innate immune features over time in BALB/c mice following low-dose *M. ulcerans* infection. (**A**) Adaptive and (**B**) innate immune features over a maximum of 19 weeks using absolute numbers of effector CD8^+^ T cells (CD8^+^ CD62L^lo^ CD44^hi^), effector CD4^+^ T cells (CD4^+^ CD62L^lo^ CD44^hi^), T_reg_ cells (CD4^+^ CD25^+^ CD62L^+^), B cells (CD19^+^ B220+), plasma cells (antibody-secreting cells) (IgD-B220^lo^ CD138+), γδ T cells (TCRγδ CD3+), NK cells (CD49b + NK1.1+), macrophages (CD64^+^ F4/80^+^), neutrophils (CD11b^+^ Ly6G^+^), and dendritic cells (CD11c^+^MHCII^+^). Error bar represents SEM. Statistically significant differences between groups are reported in Table S7 using Mann-Whitney U test. Gating strategy is shown in Fig. S1A through C. Source data are provided as a source data file. These are visualized as a heatmap (**C**) of normalized values, which do not demonstrate any clear pattern over time (BALB/c mice controls: week (W) 4–6 *n* = 5 per group, W10 *n* = 3, W > 10 *n* = 10; ~10 CFU dose: W4 – 10 *n* = 5 per group, W > 10 *n* = 10; ~100 CFU dose: W4 *n* = 4, W6–10 *n* = 5 per group, W > 10 *n* = 8).

For BALB/c mice, there were no significant differences between low-dose and high-dose mice in either adaptive (Fig. 4A) or innate (Fig. 4B) immune profiles at any timepoint. Unlike C57BL/6 mice, splenic cellular values in BALB/c mice were generally similar to controls or lower than observed in control mice, particularly 10 weeks following challenge in both high- and low-dose groups (Table S7). When values were normalized and analyzed over time; again, there was no clear pattern to emerge (Fig. 4C). These results suggest that a robust cellular response, as observed in C57BL/6 mice, may be an important factor in increased disease pathology and poorer clinical outcomes.

### High-dose challenge is associated with detection of serum anti-*M. ulcerans* antibodies

**Fig 5:**
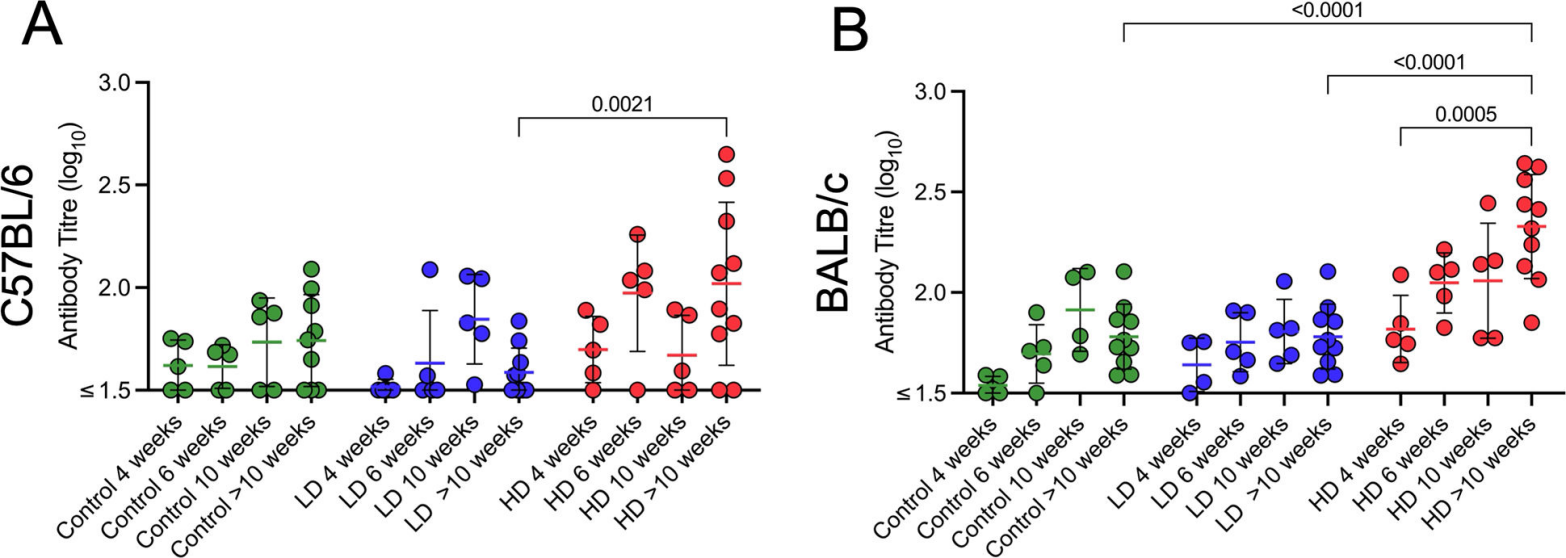
Antibody responses to low-dose (“LD”) and high-dose (“HD”) infection over time. (**A**) Antibody titers in C57BL/6 and (**B**) BALB/c serum over a maximum of 19 weeks (BALB/c) or a maximum of 15 weeks (C57BL/6) were measured by ELISA against bacterial whole-cell lysate. (C57BL/6 controls: Week (W) 4–10 *n* = 5 per group, W > 10 *n* = 9; C57BL/6 both dosing groups: W4–10 *n* = 5 per group, W > 10 *n* = 10; BALB/c controls: W4–6 *n* = 5 per group, W10 *n* = 4, W > 10 *n* = 10; BALB/c ~ 10 CFU: W4 *n* = 4, W6–10 *n* = 5 per group, W > 10 *n* = 10; BALB/c ~ 100 CFU: W4–10 *n* = 5 per group, W > 10 *n* = 10). Statistical differences were determined using two-way ANOVA. Error bars represent the SEM.

To investigate humoral immune responses induced by infection, serum from infected C57BL/6 and BALB/c mice was analyzed for antibody responses against *M. ulcerans* whole-cell lysate by ELISA. In both C57BL/6 and BALB/c mice infected with the low dose, there were no significant differences in antibody titers compared to control mice at each time point. In comparison, high-dose C57BL/6 mice that progressed to the endpoint of ulceration (>10 weeks) had significantly higher anti-*M*. *ulcerans* antibody titers than low-dose mice (Fig. 5A). High-dose BALB/c mice also had higher antibody titers at the endpoint of ulceration compared to low-dose and control mice (Fig. 5B). These data suggest that in mice, antibody responses to *M. ulcerans* are observed late in the course of infection, perhaps explained by increased extracellular bacteria at the later infection time points.

### Inflammatory responses to low-dose *M. ulcerans* in C57BL/6 mice are dominated by activated adaptive immune responses, which are associated with delayed disease progression

**Fig 6:**
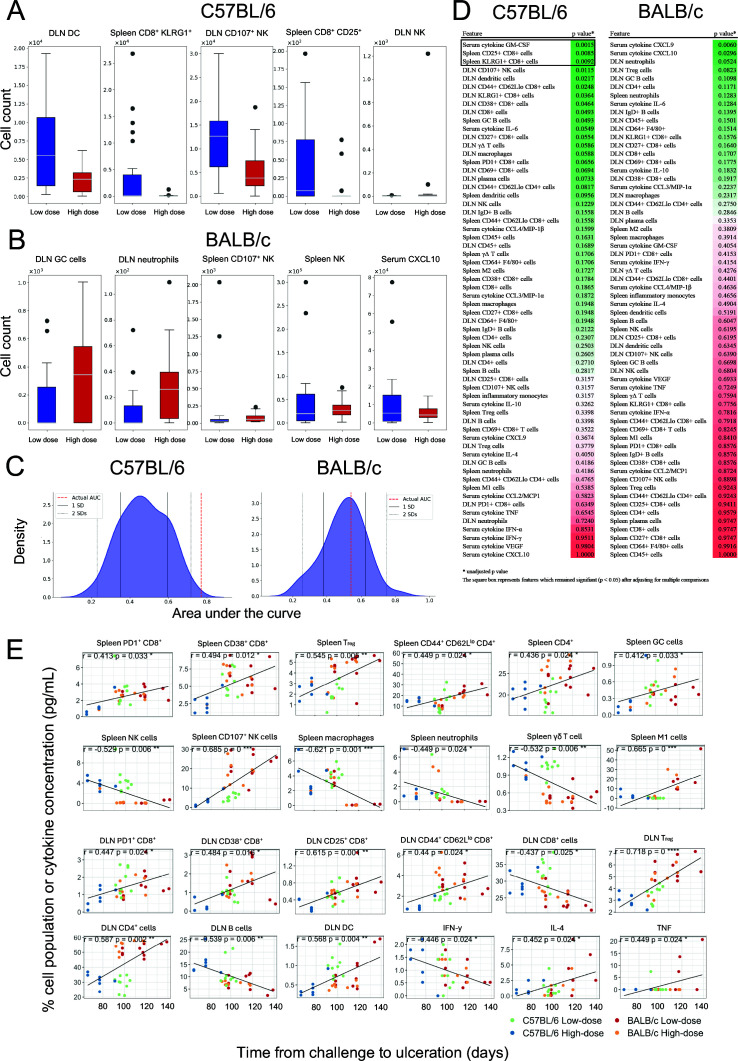
Predictive features associated with challenge “dose.” (**A**) and (**B**) demonstrate the five features ranked by importance using random forest classifiers; error bars represent SEM. (**C**) shows classifier performance demonstrated by the difference in AUC between models trained on actual data and those using randomized labels. (**D**) demonstrates the significance between high-dose and low-dose groups using univariable Mann-Whitney U tests, with *P*-value reported alongside each immunological parameter; the square box represents features which remained significant after adjusting for multiple comparisons. To adjust for false discovery due to multiple comparisons, from each group of highly correlated variables (correlation coefficient >0.8), only one representative variable was retained, and the Benjamini–Hochberg procedure was then applied; these values are also displayed in Table S9. (**E**) shows the correlation between various immune features and the time to ulceration, with statistical significance of the Pearson correlation coefficient (R) determined using Student’s t-tests, adjusting *P* values for multiple comparisons using the Benjamini-Hochberg (false discovery rate) method. (C57BL/6: ~100 CFU *n* = 7, ~10 CFU *n* = 9, BALB/c: ~100 CFU *n* = 8, ~10 CFU *n* = 10).

To define immune features associated with each respective dosing group, Random Forest classifiers were first employed to evaluate the combined ability of immune parameters and their activation markers (features) to discriminate between low- and high-dose challenge groups. This analysis identified the top five immune parameters contributing to group separation for each mouse strain, highlighting key features driving the classification. In C57BL/6 mice (Fig. 6A), this analysis highlighted the features that might be driving the inflammatory responses observed in the low-dose C57BL/6 model; most notably, DLN dendritic cells and CD107^+^ NK cells, and activated KLRG1^+^ and CD25^+^ splenic CD8^+^ cells. In BALB/c mice (Fig. 6B), activated (germinal center) DLN B cells emerged as the top predictive feature of inoculation dose. Classifier performance was evaluated by comparing the area under the curve (AUC) from the actual data to that from 100 repetitions with randomized class labels (Fig. 6C). The difference in AUC between the actual data and the randomized runs was minimal for the BALB/c group, while a significant difference, exceeding two standard deviations from the randomized mean, was observed for the C57BL/6 mice. These data confirm that robust cellular immune responses occur in C57BL/6 mice during *M. ulcerans* infection, but not in BALB/c mice. Correlation analyses of clinical and immune features confirmed these patterns (Fig S4C).

Univariate tests were also performed to evaluate differences in individual immune features between the dosing groups over the entire experimental time course (Fig. 6D). Several significant differences were observed in C57BL/6 mice. These differences were predominantly among CD8^+^ T cells and associated responses and included splenic CD25^+^ and KLRG1^+^ CD8^+^ T cells (*P* = 0.036) and serum cytokine GM-CSF (*P* = 0.020) (Table S9). No differences in immune features were observed for BALB/c mice between dose groups at any time point.

Finally, to assess whether any immunological features were positively or negatively correlated with disease progression, immune features were analyzed for each mouse strain across both doses over time (Fig. 6E). In splenic tissue, increased frequencies of immune cells correlating with delayed disease progression included activated (CD107^+^) NK cells, CD4^+^ cells, CD4^+^ effector (CD44^+^CD62L^lo^) cells, regulatory T cells, as well as activated (germinal center) B cells and CD38^+^ and PD1^+^ CD8^+^ cells. The increased splenic immune cells that correlated with more rapid disease progression included macrophages, neutrophils, and γδ T cells. In DLNs, a correlation with delayed disease progression was observed with increased presence of dendritic cells, CD4^+^ cells, regulatory T cells, as well as PD1^+^, CD38,^+^ and CD25^+^ CD8^+^ cells, and effector (CD44^+^ CD62L^lo^) CD8^+^ cells. Of note, increased total DLN CD8^+^ cell frequencies correlated with more rapid disease progression, underscoring the importance of CD8^+^ activation in delaying disease progression. Notably, although splenic macrophages were associated with disease progression, M1-biased splenic macrophages correlated with delayed disease progression. In this study, the cytokine IFN-γ was correlated with more rapid disease progression, likely influenced by the greater IFN-γ responses in mice that received the higher challenge dose, suggesting dysregulated immune responses. These analyses demonstrate the important role of adaptive immunity, and particularly activated T cells, following low-dose *M. ulcerans* infection.

## DISCUSSION

The principal goal of this study was to demonstrate the importance of accurately dosing *M. ulcerans* with a realistic bacterial inoculum and to establish a relevant animal model of BU to characterize host-pathogen responses. In Australia, *Aedes notoscriptus* mosquitoes are a vector of *M. ulcerans*, harboring a mean of ~300 total *M. ulcerans* bacteria per mosquito (range, 11–4,200 bacteria), as established by qPCR. The actual number of viable bacteria transmitted during mosquito blood-feeding is likely to be even lower than the total bacterial estimate. Here, a clear dose-response relationship in both BALB/c and C57BL/6 mice was demonstrated, with low doses extending the time to lesion onset, replicating the long incubation periods seen in humans (5, 6). Mice challenged with a lower dose also demonstrated slower progression of clinical disease, which is an important safety consideration if such dose ranges are to be used for a human challenge study, as more rapidly progressive disease is an unfavorable outcome (21, 22). Ideally, in a controlled human infection, lesions would progress slowly, as is typical in naturally occurring BU, and slower disease progression will also enable timely therapeutic intervention.

With a 100% attack rate in both groups of mice at both low and high doses, this study confirms the highly infectious nature of *M. ulcerans* in susceptible hosts. This study should encourage researchers to reframe the concept of a “high-dose” *M. ulcerans* challenge and highlight the importance of host factors on BU clinical outcomes. Compared to BALB/c mice, C57BL/6 mice demonstrated a more edematous/inflammatory phenotype, as evidenced clinically and upon detailed immunological analysis. Injecting additional *M. ulcerans* extracellular matrix alongside the bacterial inoculum may itself trigger an inflammatory response (23); the present study avoided that possibility by using a carefully prepared suspension of individual bacilli, with clumps filtered prior to cell banking (13).

The natural history of *M. ulcerans* infection involves a cycle of infection, intracellular bacillary replication, leukocyte destruction, and extracellular propagation (24). At least in C57BL/6 mice, we found that the systemic activation of cellular responses is only apparent in mice challenged with low *M. ulcerans* doses. Although the implications of this require further research, it supports the hypothesis that immune responses to vaccination may be “overwhelmed” by unrealistically high challenge doses. This will require further interrogation with promising candidate vaccinations (9) prior to low-dose challenge, particularly in C57BL/6 mice.

Reinforcing the importance of host-specific immunity, previous research demonstrated the markedly pronounced cellular response to *M. ulcerans* infection in the DLNs of C57BL/6 mice compared to BALB/c mice (23). Our study also suggests that a systemic cellular response occurs soon (~1 month) after low-dose infection, presumably promoting the recruitment of cells from the systemic circulation to the site of infection. Considering the early intracellular phase of *M. ulcerans* infection (17), it is likely that the Th1 bias of this mouse strain enables the swift recruitment of macrophages, thereby enabling a more rapid infection and inflammatory clinical phenotype. It has been assumed that phagocytes play a key role in early host defense against *M. ulcerans* (25). Counterintuitively, a strong phagocytic response may act to expose mycobacteria to susceptible cells (17), potentially enabling rapid uptake, replication, and propagation of infection. Indeed, the present study does suggest a correlation between increased systemic (i.e., early) phagocytic responses and more rapid progression of clinical disease. Co-opting the immune system is also known to occur in *M. marinum* infection, a related mycobacterium with close genetic homology (26). Intra-phagocyte trafficking of *M. ulcerans* to DLNs is observed in C57BL/6 (27) and BALB/c (28) mice and may be more likely to occur early in the course of disease, when mycolactone concentration in the surrounding tissue is low; nevertheless, due to the organism’s restricted temperature range, disease in organs might be contained by the host’s immunological response to non-viable bacilli. In the natural animal host, the native Australian possum, evidence of dissemination and cell-mediated immune control has been reported using experimental infection with *M. ulcerans* JKD8049 (29). A limitation of the present study is that we did not collect microbiological data to demonstrate systemic dissemination of infection. Previous reports have been unable to demonstrate the presence of *M. ulcerans* in the spleens of C57BL/6 mice (27), although antigen presentation within lymphoid tissues is not unexpected (30). Nevertheless, here we have shown that a robust, early, systemic, Th1-biased cellular immune response is unable to protect C57BL/6 mice from challenge with a low number of *M. ulcerans* bacilli.

This study demonstrated the important role of specific cellular responses to low-dose *M. ulcerans* infection. In the low-dose C57BL/6 model, it appears that early splenic cellular responses are broad, including both innate and adaptive immune cells. Univariate and machine learning analyses showed that immune responses to low-dose infection are dominated by activated CD8^+^ T-cell responses, and correlation models show that activated CD8^+^ T cells in spleen and DLN are associated with a delayed disease course (Fig. 6). Future applications of this model should aim to confirm this finding and further characterize CD8^+^ antigen specificity. The specific mechanisms by which CD8^+^ T cells fail to protect from ulceration remain to be understood. Investigating the mechanism(s) of this failure has potentially important implications for creating effective vaccination strategies, such as building vaccines that improve antigenic presentation and stimulate *M. ulcerans-*specific CD8^+^ T-cell responses. For instance, saponin-based adjuvants strongly induce cross-presentation by dendritic cells, leading to subsequent CD8^+^ T-cell activation (31, 32). Using such adjuvants with promising subunit vaccine candidates may offer additional protection from infection via antigen-specific CD8^+^ T-cell responses and warrant consideration in future research.

Dendritic cells also have a prominent role in low-dose *M. ulcerans* infection, with more dendritic cells observed in the DLNs of C57BL/6 mice later in the course of disease. DLN dendritic cells appeared significant in univariate analysis, although this difference just exceeded the statistical significance threshold when correcting for multiple comparisons (*P* = 0.050). Dendritic cells were the top classifier feature explaining the difference between the high- and low-dose models. Finally, DLN dendritic cells were also associated with delayed disease progression, highlighting the important role of these key antigen-presenting cells in low-dose *M. ulcerans* infection.

Our data suggest that a systemic reduction in immune cellularity occurs in C57BL/6 mice as bacterial burden increases over time (Fig. 2A). This phenomenon was not observed in our study in BALB/c mice, nor in previous research (28). It remains unclear whether this reduction reflects true systemic immune suppression in C57BL/6 mice, or regional sequestration of cells at the site of infection. Considering the remarkably edematous/inflammatory nature of the infection locoregionally, the latter is favored to be the likely mechanism. As reported by Converse and colleagues (33), we found that *M. ulcerans* growth kinetics were similar in both BALB/c and C57BL/6 mice across all timepoints, so any differences observed between each mouse strain are unlikely to be influenced by host control of bacterial growth or differential mycolactone concentration. Nevertheless, a limitation of this study is that mycolactone concentrations were not measured; therefore, we cannot exclude the influence of potential variable mycolactone expression over time or between each mouse strain.

Another limitation of this research is that very early immune responses (<4 weeks) were not captured. Oliveira et al. previously demonstrated acute neutrophilic infiltration at the site of inoculation in BALB/c mice, although their study used a very large inoculum (1 × 10^5^ AFB) of a virulent African (mycolactone A/B-producing) isolate, which resulted in leukocyte destruction as early as 24 hours after inoculation (24). The authors also showed that inoculation with ~316 AFB had a similar histopathological outcome with delayed kinetics, which was reinforced by the present study. Nevertheless, neutrophils were not observed in tail histology at any of our earliest timepoints. A previous BALB/c mouse model of infection by Fraga et al*.* demonstrated early accumulation of CD4^+^ lymphocytes in DLN tissue (around 14 days post-challenge). Their study is difficult to compare and may be less generalizable, as ulceration occurred within 24 days of challenge, compared to an average of 116 days in BALB/c mice in our low-dose challenge model. They also used a very high inoculation dose of a virulent African isolate (3 × 10^5^ AFB) using a footpad site of inoculation (28).

Although the murine footpad model of BU has been utilized extensively in previous vaccine research (9), the footpad model has numerous limitations. Footpad infection more readily exposes lesions to the environment, which may increase the risk of irritation and superinfection, confounding markers of inflammation. Similar to the footpad site, the tail model shares the convenience of little hair coverage. The tail also appears a favored site for the development of BU lesions, even in mice inoculated intracerebrally (34). As the foot is a weight-bearing structure, mice may develop gait abnormalities, and although pain is uncharacteristic for this disease, the confined anatomical space in the footpad may predispose mice to discomfort associated with tissue swelling. Previous studies have identified the hock as an ethically acceptable alternative to the footpad model (23), although the tail was not specifically characterized, and the high *M. ulcerans* dose (~10^7^ CFU) was based on the wet weight of injected material, which is inherently less accurate than the enumeration method reported in the present study. The hock infection model is also impractical for histopathology, as bacilli are difficult to localize, and macroscopically detectable inflammation is not observed (23). An additional practical benefit of the tail model is that it is easier to inspect during routine observation. The tail is also a reproducible site of infection, as the incubation period in naïve BALB/c mice receiving a low-dose challenge in the present study was identical to our initial pilot model using *M. ulcerans* JKD8049 from the same cell bank used in the present study (15). The refined tail model presented here today, therefore, represents an ethically acceptable, reproducible, and convenient site for future murine BU research.

Although we used BALB/c and C57BL/6 mice to understand the infection rate in mammalian hosts with broad immune biases, we are unable to predict whether this will similarly translate to a high infection rate in a human model of infection. Similar to our study, Coutanceau et al. did not observe granuloma formation in either BALB/c or C57BL/6 mice (27), which is unlike human cases, where granulomas are associated with healing (14). Genetic differences between inbred laboratory mice and humans are likely to confer varying degrees of susceptibility to infection. For example, both BALB/c and C57BL/6 mice are known to contain polymorphisms in the natural resistance-associated macrophage protein gene (*NRAMP1*) (35); in humans, polymorphisms in this gene are also associated with increased susceptibility to *M. ulcerans* infection, although the majority of individuals selected for challenge are unlikely to carry these polymorphisms (36).

### Conclusion

By analyzing an infection time course of over 70 clinical, immunological, and microbiological parameters, this study identified dose-related differences in outcomes following *M. ulcerans* infection in mice. We observed that strong cell-mediated responses were associated with more rapid Buruli ulcer onset. In C57BL/6 mice, low bacterial challenge doses trigger an early systemic immune response, well before the onset of clinical disease, enabling the trafficking of immune cells to the site of infection while the infection develops. In the higher dose challenge model, these immunological features were likely masked by higher bacterial concentrations and (presumably) more rapid mycolactone accumulation. Antibody responses were not evident following low-dose infection, and antibody presence after high-dose infection did not predict disease protection. Given these findings and considering recent evidence suggesting that low inocula are likely acquired during natural infection, we suggest that future pre-clinical research considers using low challenge doses. In the context of a planned controlled human infection of *M. ulcerans*, this study supports lower challenge doses that will slow the course of clinical disease. A reliable, slowly progressing infection is preferred to a rapid-onset disease phenotype. Vaccination approaches that facilitate CD8^+^ T-cell activation in response to *M. ulcerans* infection may facilitate protection from disease.

## Data Availability

All primary data not included in the paper or supplemental files are available at https://figshare.com/s/2c9befed57e953f61a63.

## SUPPLEMENTAL MATERIAL

Supplemental material mbio.01931-25-s0001.pdf Supplemental figures and tables.

ASM does not own the copyrights to Supplemental Material that may be linked to, or accessed through, an article. The authors have granted ASM a non-exclusive, world-wide license to publish the Supplemental Material files. Please contact the corresponding author directly for reuse.
