# Supplementary material for: A refined low-dose murine model of Mycobacterium ulcerans infection to assess integrated immune networks in Buruli ulcer pathogenesis
Source: mBio. 2025 Aug 18;16(9):e01931-25. doi: 10.1128/mbio.01931-25 (PMC12421887; doi:10.1128/mbio.01931-25)
Supplement: Supplemental material — Supplemental figures and tables. [file mbio.01931-25-s0001.pdf]

Supplementary material for:

A refined low-dose murine model of *Mycobacterium ulcerans* infection to assess integrated immune networks in Buruli ulcer pathogenesis

Stephen Muhi, Isabelle J.H. Foo, Lukasz Kedzierski, Jessica L. Porter, Hayley A. McQuilten, Brian Howden, Katherine Kedzierska, Andrew H. Buultjens, Brendon Y. Chua and Timothy P. Stinear

Table of contents:

Figures **Page No.**

Figure S1. Gating strategy for flow cytometry. **2**

Figure S2. Microbiological and clinical features over time. **3**

Figure S3. Microbiological correlates of clinical disease over time. **4**

Figure S4. Differences in cytokines and chemokines **5**

Tables

Table S1. Clinical, microbiological and histopathological features: C57BL6 mice, high dose. **6**

Table S2. Clinical, microbiological and histopathological features: C57BL6 mice, low dose. **8**

Table S3. Clinical, microbiological and histopathological features: BALB/c mice, high dose. **9**

Table S4. Clinical, microbiological and histopathological features: BALB/c mice, low dose. **11**

Table S5. Clinical and biometric features of C57BL/6 mice with lesions, and representative controls. **13**

Table S6. Clinical and biometric features of BALB/c mice with lesions, and representative controls. **14**

Table S7. Differences between *M. ulcerans* dosing groups (high dose, low dose, and control) and various immune features over time (unadjusted univariate analysis). **15**

Table S8. p values reaching statistical significance following correction for multiple analyses across the entire univariate dataset. C57BL/6 mice only are presented. **16**

Table S9. p values reaching statistical significance following correction for multiple analyses across the dataset presented in Fig 5E. C57BL/6 mice only are presented. **16**

References **17**

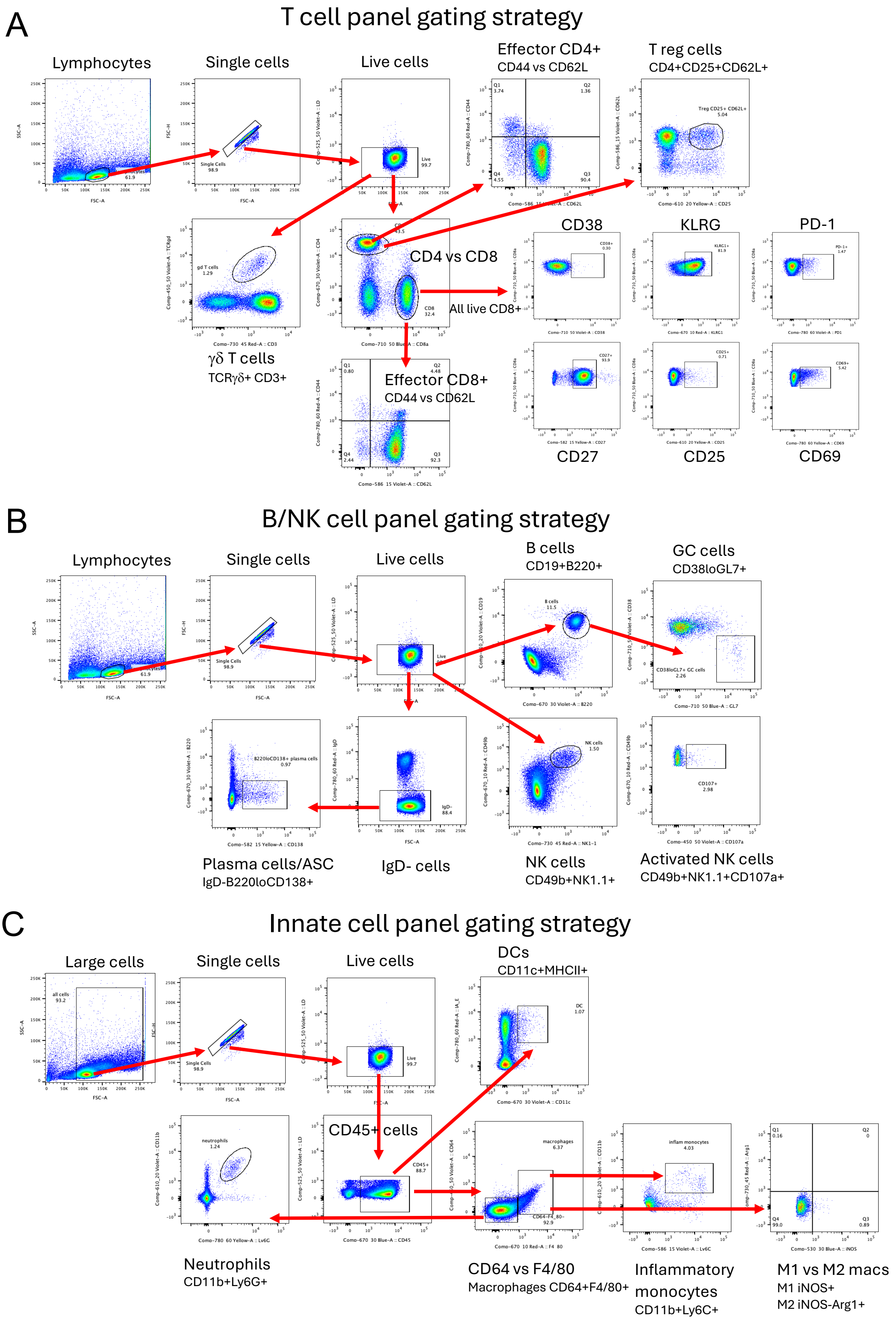

Figure S1. **Gating strategy for flow cytometry**. **A** T cells, **B** B/NK cells and **C** innate immune cells.

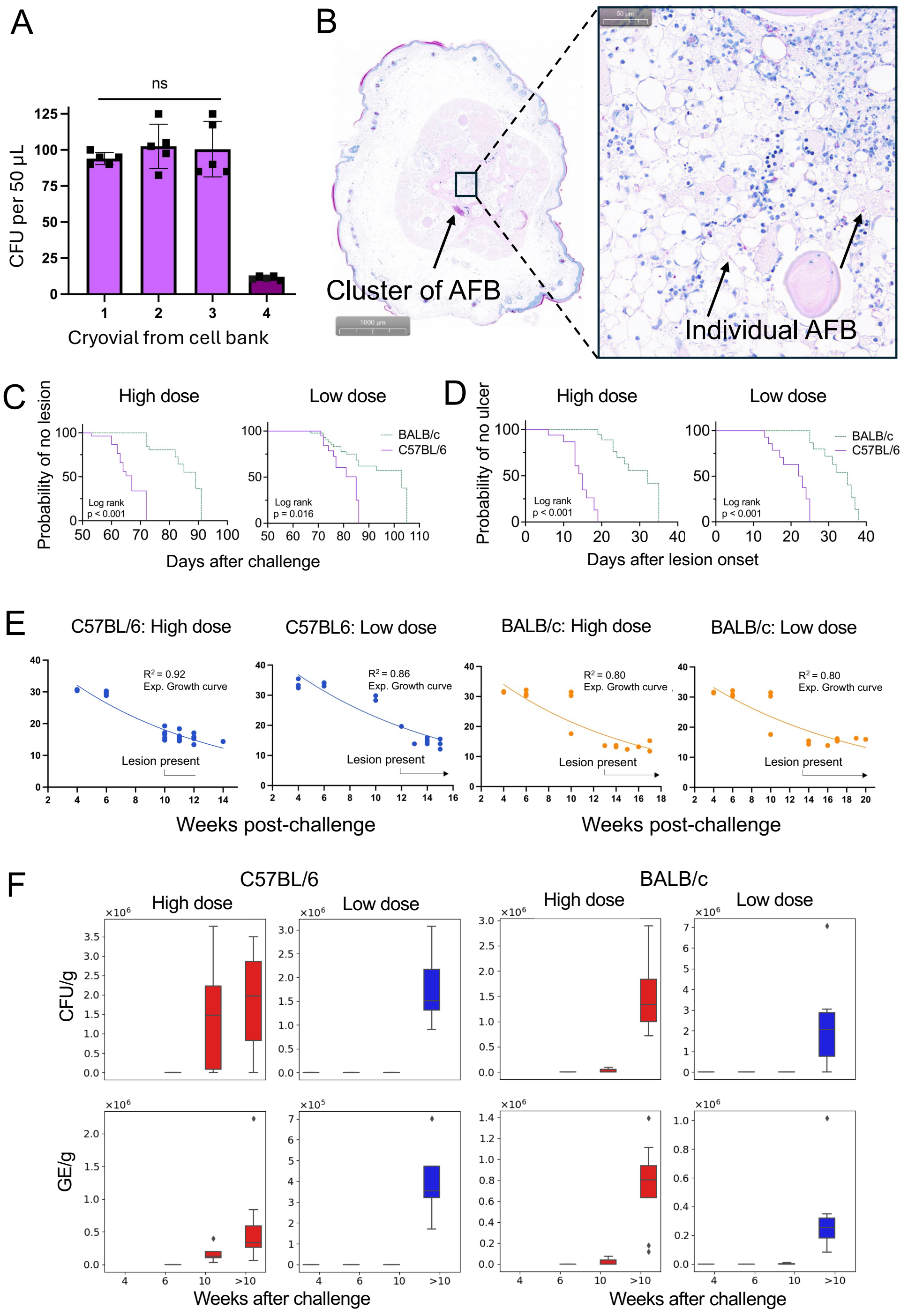

Figure S2. Microbiological and clinical features over time. A CFU challenge dose enumeration as calculated from each vial prior to inoculation; the three columns on the left show the CFU count in the vials used for high dose challenge. The column on the far right illustrates the mean CFU value injected after dilution to a low dose. Errors bars represent 95% confidence interval. **B** Cross section through the ulcerated tail of a C57BL/6 mouse (#HB8.5) challenged with ~ 100 CFU of *M. ulcerans* JKD8049 and stained with Ziehl–Neelsen stain; there is a large and dense clump of *M. ulcerans* acid fast bacilli (AFB) immediately adjacent to the vertebral bone; inset demonstrates individual AFB visible within the bone marrow tissue (inset bar represents 50 µm length at magnification of 20x). **C** survival curves demonstrating the time to lesion onset in C57BL/6 mice compared to BALB/c mice (~10 CFU challenge dose: C57BL6 n=10, BALB/c n=10; ~100 CFU challenge dose: C57BL6 n=15, BALB/c n=10). **D** survival curves demonstrating the time from lesion onset to ulceration in C57BL/6 mice compared to BALB/c mice (~10 CFU challenge dose: C57BL6 n=10, BALB/c n=10; ~100 CFU challenge dose: C57BL6 n=12, BALB/c n=10). **E** visualises *M. ulcerans* IS*2404* PCR cycle threshold over time according to dose and mouse type (~10 CFU dose C57BL/6: week (W) 4 n=3, W6 n=3, W10 n=3, W>10 n=10; ~100 CFU dose, C57BL/6: W4 n=3, W6 n=3, W10 n=5, W>10 n=10; ~10 CFU dose, BALB/c: W4 n=3, W6 n=3, W10 n=3, W>10 n=10; ~100 CFU BALB/c: W4 n=3, W6 n=3, W10 n=3, W>10 n=10). The line represents the exponential growth curve. R^2 indicates the goodness-of-fit. **F** microbiological enumeration (in CFU/g and GE/g) of bacilli over time in both low and high dose cohorts, error bars represents SEM.

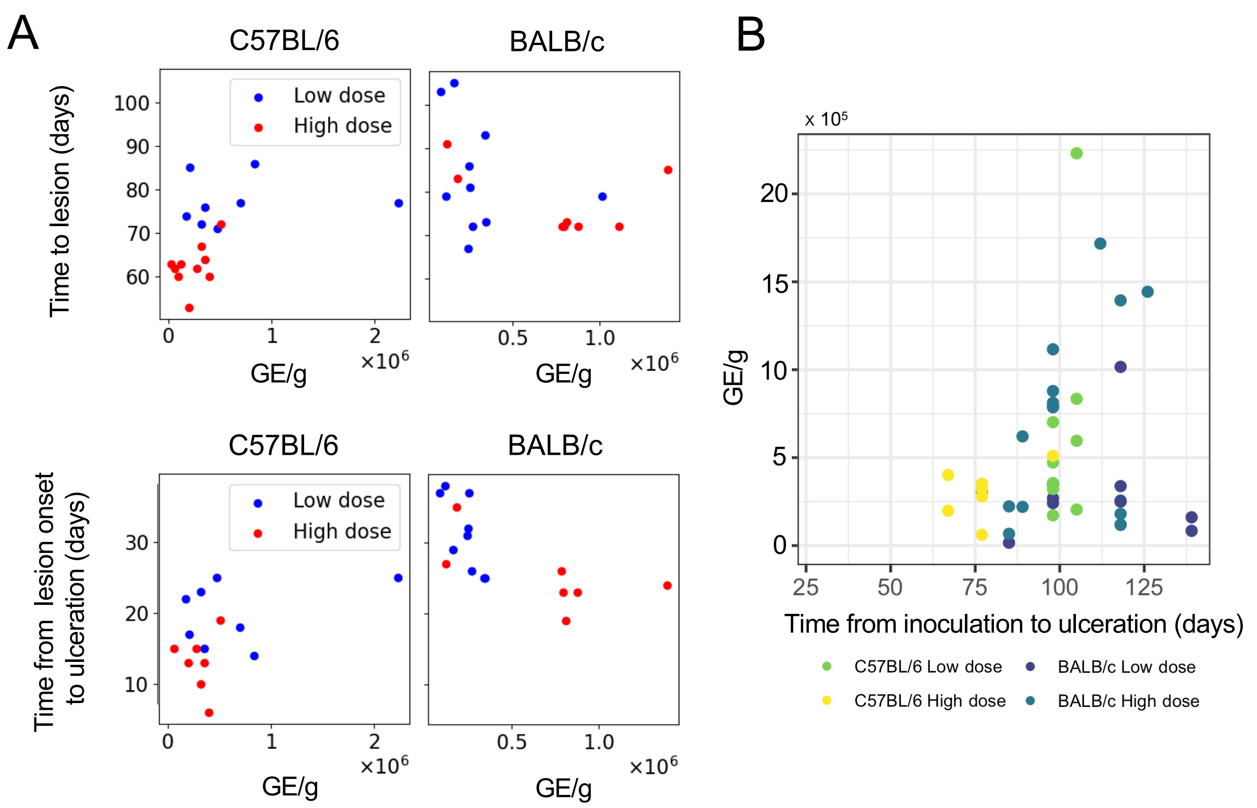

Figure S3. **Microbiological correlates of clinical disease over time**. **A** the relationship between clinical features of infection (time to lesion onset and time from lesion onset to ulceration) and correlation with the burden of bacilli in GE/g. **B** correlation plot between GE/g and time from inoculation to ulceration across both mouse lines and dosing groups.

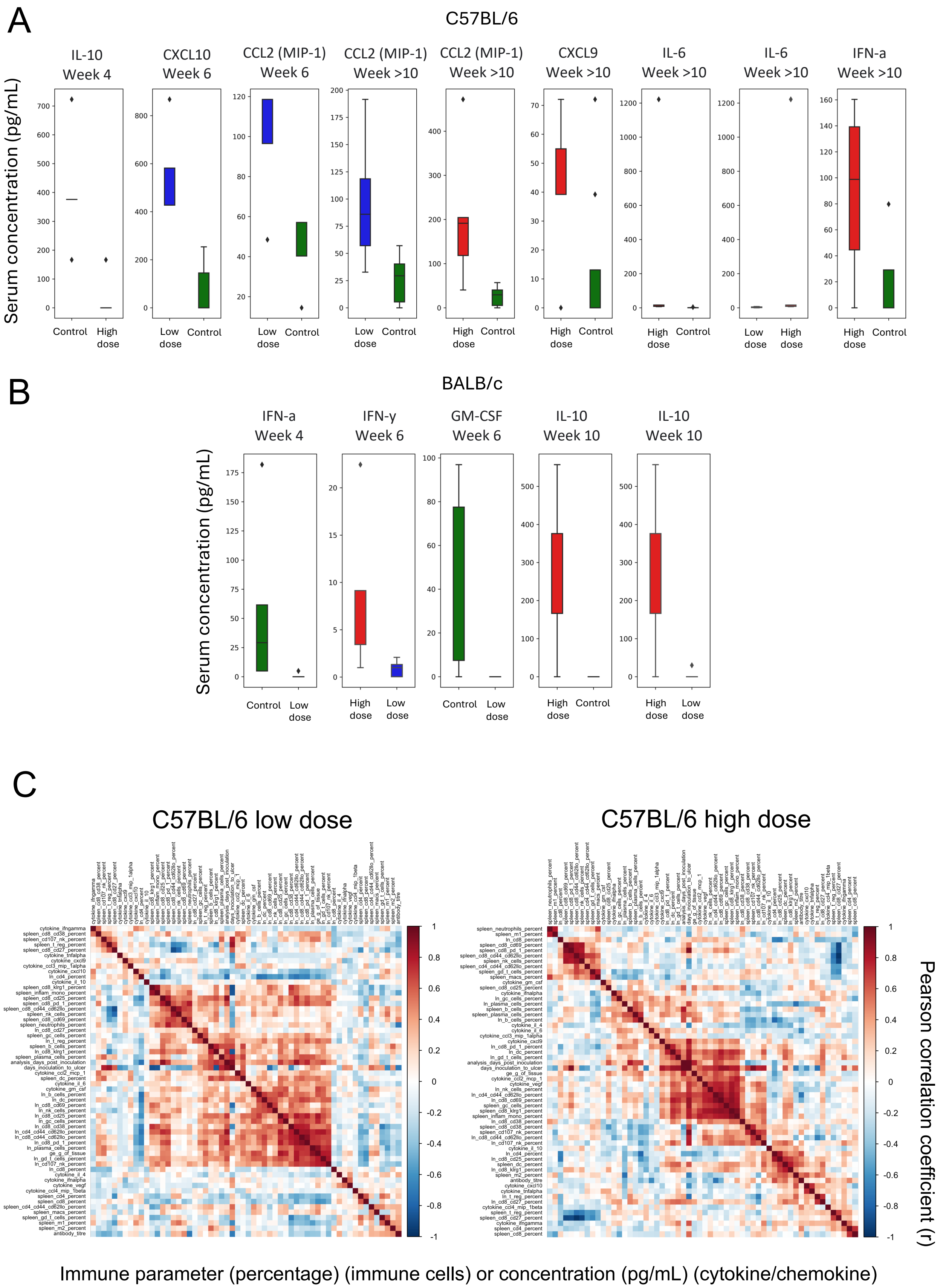

Figure S4. Differences in cytokines and chemokines A C57BL/6 mice and **B** BALB/c mice; error bar represents the SEM. **C** correlation matrix of clinical and immune features over time; dark red (Pearson correlation coefficient = 1) suggests a strong correlation, dark blue (Pearson correlation coefficient = -1) suggests a strong negative correlation.

Table S1. Clinical, microbiological and histopathological features: C57BL6 mice, high dose.

Time after challenge (weeks)	Mean IS*2404* PCR Ct	GE /g of tissue	Lesion	Lesion type	CFU/g tissue	Histology report
4	N/A	N/A	No	N/A	N/A	No lesions seen.
4	N/A	N/A	No	N/A	N/A	No lesions seen.
4	30.73	84.53	No	N/A	No growth	N/A
4	30.73	65.75	No	N/A	No growth	N/A
4	30.37	98.43	No	N/A	No growth	N/A
6	N/A	N/A	No	N/A	N/A	No lesions seen.
6	N/A	N/A	No	N/A	N/A	No lesions seen.
6	30.28	77.66	No	N/A	No growth	N/A
6	29.63	100.11	No	N/A	152.0	N/A
6	28.85	206.15	No	N/A	119.00	N/A
10	17.13	1.01E+05	Yes	Pre-ulcerative	1.47E+06	Multifocal dermal and subcutaneous oedema, necrosis, moderate macrophage and lymphocyte infiltrations. Necrosis associated with AFB.
10	14.81	4.01E+05	Yes	Ulcer	2.23E+06	Mild dermal and moderate subcutaneous oedema, mild necrosis, infiltrations of macrophages and lymphocytes. AFB present in one area.
10	19.28	2.68E+04	Yes	Pre-ulcerative	No growth	Moderate to severe multifocal to diffuse dermal and subcutaneous oedema with scanty macrophages (occasionally with scanty AFB in the cytoplasm) and neutrophil infiltration. Destruction of blood vessel walls and skeletal muscle and tendon necrosis, areas associated with multifocal abundant acid-fast bacilli. No epidermal necrosis.
10	16.63	1.25E+05	Yes	Pre-ulcerative	7.88E+04	Moderate multifocal to diffuse dermal and subcutaneous oedema, vasculopathy and infiltration of macrophages, lymphocytes, neutrophils. Multifocal skeletal myofiber necrosis with multifocal AFB.
10	15.55	1.97E+05	Yes	Ulcer	3.76E+06	Severe multifocal full-thickness epidermal necrosis, multifocal epidermal pustule formation and diffuse subcutaneous oedema, with infiltrations of macrophages, lymphocytes, neutrophils. There is vasculitis, and tissue necrosis including skeletal myofiber necrosis with multifocal AFB.
11	16.11	3.06E+05	Yes	Ulcer	5.67E+05	Severe locally extensive full-thickness epidermal necrosis, multifocal epidermal vesicles, diffuse subcutaneous oedema, multifocal vasculopathy, infiltrations of macrophages, lymphocytes, neutrophils. Multifocal necrosis of connective tissue and skeletal muscle with multifocal AFB.
11	18.37	6.14E+04	Yes	Ulcer	2.17E+06	Severe locally extensive full-thickness epidermal necrosis, diffuse dermal and subcutaneous oedema, multifocal vasculopathy, infiltrations of macrophages, lymphocytes, neutrophils. Multifocal necrosis of connective tissue, skeletal muscle and bone marrow with multifocal AFB.
11	15.50	3.51E+05	Yes	Ulcer	1.10E+06	Severe locally extensive full-thickness necrosis of the epidermis. The intact epidermis shows multifocal hyperplasia and overlies markedly oedematous dermis and subcutaneous with infiltrations of inflammatory cells, macrophages, lymphocytes, neutrophils; tissue necrosis, vasculopathy and necrosis of skeletal muscles. AFB are scattered throughout the oedematous and necrotic tissue in low numbers.
11	15.08	2.79E+05	Yes	Ulcer	1.77E+06	Severe locally extensive full-thickness necrosis of the epidermis. The intact epidermis shows multifocal hyperplasia. Severe multifocal to coalescing dermal and subcutaneous oedema with infiltrations of inflammatory cells, macrophages, lymphocytes, neutrophils; tissue necrosis, vasculopathy and necrosis of skeletal muscles. AFB are present scattered and within necrotic tissue.
11	14.54	3.20E+05	Yes	Ulcer	3.50E+06	Focal epidermal erosion overlying an area of mild subcutaneous oedema, inflammation with scanty acid-fast bacilli. Moderate to severe extensive dermal and subcutaneous oedema, multifocal vasculopathy, infiltrations of macrophages, lymphocytes, neutrophils. Multifocal necrosis of connective tissue and skeletal muscle with multifocal AFB.
12	15.27	2.23E+05	Yes	Ulcer	4.45E+05	Focal epidermal erosion, multifocal hyperplasia and diffuse hyperkeratosis, severe segmental subcutaneous oedema, mixed inflammatory cell infiltrates multifocal necrosis of skeletal muscle and bone marrow, and focal vasculitis. AFB associated with necrotic tissue and within the wall of the inflamed blood vessels.

12	15.83	2.20E+05	Yes	Ulcer	1.35E+06	Multifocal full thickness epidermal necrosis. Moderate diffuse subcutaneous oedema with macrophage, neutrophils, lymphocytes, multifocal tissue necrosis, skeletal muscle and into the bone marrow. Multifocal AFB associated with necrosis and in the bone marrow.
12	13.39	6.21E+05	Yes	Ulcer	2.66E+06	Severe diffuse subcutaneous oedema, inflammatory infiltrates (macrophages, lymphocytes, neutrophils), vasculopathy, tissue necrosis with AFB. Multifocal epidermal hyperplasia, hyperkeratosis and focal pustule formation.
12	17.09	6.71E+04	Yes	Ulcer	1.54E+04	Moderate to severe multifocal to diffuse dermal and subcutaneous oedema with scattered macrophages, lymphocytes and neutrophils associated with multifocal AFB. Focal vasculopathy.
14	14.38	5.10E+05	Yes	Ulcer	2.83E+06	Severe full thickness skin and subcutaneous necrosis of >60% of the section with deep ulceration and loss of adnexal structures. Intense inflammatory infiltrates of neutrophils, macrophages, lymphocytes are present. Severe vasculopathy associated with connective tissue and skeletal muscle necrosis. Severe subcutaneous oedema of the remaining section with multifocal necrosis and skeletal muscle necrosis and moderate mixed inflammatory infiltrates. Multiple clusters of AFB in affected areas.

Table S2. Clinical, microbiological and histopathological features: C57BL6 mice, low dose.

Time after challenge (weeks)	Mean IS*2404* PCR Ct	GE /g of tissue	Lesion	Lesion type	CFU/g tissue	Histology report
4	N/A	N/A	N/A	N/A	N/A	N/A
4	N/A	N/A	No	N/A	N/A	No lesions seen.
4	33.32	14.06	No	N/A	No growth	N/A
4	32.43	28.98	No	N/A	No growth	N/A
4	35.46	4.42	No	N/A	No growth	N/A
6	N/A	N/A	No	N/A	N/A	No lesions seen.
6	N/A	N/A	No	N/A	N/A	No lesions seen.
6	33.01	16.39	No	N/A	No growth	N/A
6	33.96	7.59	No	N/A	No growth	N/A
6	34.15	7.38	No	N/A	No growth	N/A
10	N/A	N/A	No	N/A	N/A	Mild subcutaneous oedema, scanty lymphocytes, no pathogens.
10	N/A	N/A	No	N/A	N/A	Mild focal subcutaneous haemorrhage and oedema. Scanty scattered lymphocytes. No pathogens
10	28.28	171.27	No	N/A	5.10E+03	N/A
10	28.28	130.82	No	N/A	No growth	N/A
10	29.84	39.44	No	N/A	3.67E+03	N/A
12	19.66	1.62E+04	Yes	Ulcer	No growth	Missing data
13	13.85	3.55E+05	Yes	Ulcer	3.07E+06	Mild dermal and moderate subcutaneous oedema, mononuclear cell infiltrates, multifocal skeletal muscle necrosis. Scattered AFB present in necrotic and oedematous areas.
14	14.35	7.02E+05	Yes	Ulcer	9.03E+05	Severe subcutaneous oedema, mild inflammatory cell infiltrates (macrophages, neutrophils, lymphocytes), multifocal connective tissue and skeletal muscle necrosis. Locally extensive area of full thickness necrosis and ulceration of the epidermis. Clusters and single AFB are present.
14	15.02	3.22E+05	Yes	Ulcer	2.17E+06	Moderate dermal and severe subcutaneous oedema with focal vasculopathy, mononuclear cell infiltrates and connective tissue and skeletal muscle necrosis and loss. Moderate numbers of AFB scattered within necrotic and oedematous tissues. Mild diffuse epidermal hyperplasia.
14	13.84	8.34E+05	Yes	Ulcer	2.94E+06	Multifocal epidermal erosion with serocellular crusting. Moderate dermal and subcutaneous oedema, mixed inflammatory infiltrates, multifocal necrotising vasculitis and multifocal necrosis of skeletal muscle sand interstitial tissues. Multifocal clusters of AFB in necrotic tissues and blood vessels and scattered within the oedematous subcutaneous tissues.
14	14.38	4.72E+05	Yes	Ulcer	1.51E+06	Large artefact with loss of subcutaneous tissues. Severe subcutaneous oedema, locally extensive epidermal and dermal necrosis, 50% of the perimeter. Severe focal necrotising vasculitis. Multifocal subcutaneous and skeletal muscle necrosis. Macrophage, neutrophil and lymphocyte infiltrations. multifocal abundant clusters and scattered AFB.
14	15.91	1.72E+05	Yes	Ulcer	1.31E+06	Multifocal dermal and diffuse subcutaneous oedema, with mixed inflammatory cell infiltrates, severe vasculopathy associated with necrosis. Necrosis of scattered skeletal muscle fibres and clusters and scattered AFB.
15	13.84	5.96E+05	Yes	Ulcer	3.54E+06	Moderate to diffuse dermal and subcutaneous oedema with moderate mixed inflammatory cell infiltrations, macrophages, and lymphocytes, with scattered intra-cellular and interstitial AFB. Multifocal vasculopathy. Multifocal skeletal muscle necrosis.
15	12.08	2.23E+06	Yes	Ulcer	No growth	Moderate dermal and subcutaneous oedema and vasculopathy with multifocal skeletal muscle and interstitial necrosis. Infiltrations of mixed inflammatory cells, macrophages and lymphocytes. AFB within the necrotic foci and associated with vasculitis and subcutaneous tissues.
15	15.44	2.05E+05	Yes	Ulcer	No growth	Severe full-thickness epidermal necrosis and erosion. Severe diffuse subcutaneous oedema with inflammatory cell infiltrates with scattered acid-fast bacilli. Focal severe necrotising vasculitis with abundant AFB. Multifocal skeletal muscle and interstitial necrosis with clusters of AFB.

Table S3. Clinical, microbiological and histopathological features: BALB/c mice, high dose.

Time after challenge (weeks)	Mean IS*2404* PCR Ct	GE /g of tissue	Lesion	Lesion type	CFU/g tissue	Histology report
4	N/A	N/A	No	N/A	N/A	1 focus of skeletal muscle bundle hypertrophy, incidental.
4	N/A	N/A	No	N/A	N/A	No lesions seen.
4	31.66	44.75	No	N/A	No growth	N/A
4	31.62	41.93	No	N/A	No growth	N/A
4	31.34	57.68	No	N/A	No growth	N/A
6	N/A	N/A	No	N/A	N/A	No lesions seen.
6	N/A	N/A	No	N/A	N/A	No lesions seen.
6	30.28	81.66	No	N/A	No growth	N/A
6	32.15	25.57	No	N/A	No growth	N/A
6	30.81	52.67	No	N/A	No growth	N/A
10	N/A	N/A	No	N/A	N/A	No lesions seen.
10	N/A	N/A	No	N/A	N/A	No lesions seen.
10	30.27	30.38	No	N/A	2.00E+03	N/A
10	17.60	7.45E+04	No	N/A	1.00E+05	N/A
10	31.48	19.11	No	N/A	2.56E+02	N/A
13	13.63	8.12E+05	Yes	Ulcer	2.67E+06	Marked subcutaneous oedema and multifocal epidermal ulceration and segmental epidermal erosion, hyperplasia and hyperkeratosis. Focal epidermal pustule. Multifocal necrosis of skeletal muscles and connective tissues. Moderate mononuclear cell infiltration. Multifocal AFB, mostly focused in necrotic areas but also identified in the bone marrow.
14	13.23	1.12E+06	Yes	Ulcer	7.18E+05	Severe diffuse dermal and subcutaneous oedema, multifocal subcutaneous and muscle necrosis, infiltrations of macrophages, lymphocytes, neutrophils. Multifocal AFB, including in a follicle.
14	13.66	7.95E+05	Yes	Ulcer	2.89E+06	Moderate to severe diffuse dermal and subcutaneous oedema, necrosis of skeletal muscles and subcutaneous connective tissues, mononuclear cell infiltrates and AFB present.
14	13.19	8.79E+05	Yes	Ulcer	1.56E+06	Moderate to severe segmental dermal and subcutaneous oedema with multifocal necrosis and necrosis of the skeletal muscle. Focal epidermal ulceration. Mononuclear cell infiltrates. Diffuse epidermal hyperplasia and hyperkeratosis. Multifocal aggregates of AFB and scattered single bacilli within subcutaneous oedema tissue.
14	13.68	7.87E+05	Yes	Ulcer	1.22E+06	Severe diffuse dermal and subcutaneous oedema, mononuclear cell infiltrates, epidermal hyperplasia and hyperkeratosis and necrosis of skeletal muscle and connective tissues. Numerous AFB in areas of necrosis and scattered in the subcutaneous tissue.
15	12.38	1.72E+06	Yes	Ulcer	2.53E+06	Marked epidermal ulceration-transmural necrosis with pustule formation. Moderate segmental epidermal hyperplasia and hyperkeratosis. Severe diffuse dermal and subcutaneous oedema with mixed inflammatory infiltrates of macrophages and lymphocytes. Multifocal mild to severe vasculopathy with focal necrotising leukocytoclastic vasculitis, associated with myriad of AFB. Multifocal necrosis with cellular debris and the presence of AFB.
16	13.21	1.39E+06	Yes	Ulcer	7.67E+05	Segmental epidermal hyperplasia and hyperkeratosis. Mild dermal and moderate to severe subcutaneous oedema, multifocal necrotising vasculitis with tissue necrosis and mixed inflammatory infiltrates. Multifocal skeletal muscle fibre necrosis and oedema. AFB are present in clusters in necrotic tissue, within affected blood vessels perivascular tissues, vascular wall and lumen, and scattered in the subcutaneous tissue.
17	15.92	1.19E+05	Yes	Ulcer	1.07E+06	Severe diffuse dermal and subcutaneous oedema, mild mixed inflammatory cell infiltrate, multifocal vasculopathy, multifocal necrosis of the subcutaneous connective tissue and skeletal muscles fibres.

17	15.24	1.80E+05	Yes	Ulcer	1.45E+06	Severe segmental, (50%) dermal and subcutaneous oedema with multifocal tissue necrosis and inflammatory cell infiltration. Focal large cluster of AFB in the necrotic tissue.
17	11.78	1.44E+06	Yes	Ulcer	8.26E+06	Severe locally extensive full-thickness epidermal necrosis with serocellular crusting and multifocal pustules. Severe subcutaneous oedema with moderate mixed neutrophil, lymphocyte and macrophage infiltrations and vasculopathy. Large foci of skeletal muscle and interstitial necrosis. Numerous clusters of AFB in association with necrotic tissues and within inflammatory cells in the subcutaneous oedema.

Table S4. Clinical, microbiological and histopathological features: BALB/c mice, low dose.

Time after challenge (weeks)	Mean IS*2404* PCR Ct	GE /g of tissue	Lesion	Lesion type	CFU/g tissue	Histology report
4	N/A	N/A	No	N/A	N/A	Incidental tendon degeneration, tendinosis. No pathogens.
4	N/A	N/A	No	N/A	N/A	Incidental tendon degeneration, tendinosis. No pathogens.
4	37.42	1.32	No	N/A	No growth	N/A
4	31.78	39.25	No	N/A	No growth	N/A
4	33.35	17.15	No	N/A	No growth	N/A
6	N/A	N/A	No	N/A	N/A	Mild focal subcutaneous mononuclear cell infiltration into adipose tissue. No other pathology. No pathogens.
6	N/A	N/A	No	N/A	N/A	No lesions seen.
6	33.51	10.38	No	N/A	No growth	N/A
6	33.92	8.13	No	N/A	No growth	N/A
6	33.56	12.22	No	N/A	No growth	N/A
10	N/A	N/A	No	N/A	N/A	No lesions seen.
10	N/A	N/A	No	N/A	N/A	There is loss of fascicle structures, not sure what the pathogenesis of this change is. No pathogens. Several artefacts in these tendons/fascicles.
10	29.38	67.46	No	N/A	4.36E+03	N/A
10	29.98	54.38	No	N/A	No growth	N/A
10	21.76	8.63E+03	No	N/A	5.88E+02	N/A
14	15.34	2.42E+05	Yes	Ulcer	2.38E+06	Severe diffuse dermal and subcutaneous oedema with mild macrophage, lymphocyte, neutrophil infiltrations. Focal vasculopathy. Multifocal necrosis of skeletal muscle and connective tissue. Numerous foci of AFB.
14	15.46	2.69E+05	Yes	Ulcer	2.78E+05	Moderate diffuse subcutaneous oedema with mild inflammatory infiltrates, multifocal muscle necrosis and oedema. Multifocal AFB. Multifocal epidermal hyperplasia and hyperkeratosis.
14	14.36	3.48E+05	Yes	Ulcer	1.76E+06	Moderate diffuse subcutaneous oedema, scant inflammatory cell infiltration, vasculopathy, necrosis of connective tissue and skeletal muscle. Multifocal epidermal hyperplasia and hyperkeratosis. Multifocal AFB.
16	13.93	1.02E+06	Yes	Ulcer	7.06E+06	Severe locally extensive full-thickness necrosis and inflammation of the epidermis, dermis and subcutaneous tissues. Diffuse dermal and subcutaneous oedema with vasculopathy and mixed inflammatory infiltrates. Multifocal deep subcutaneous and skeletal muscle necrosis and inflammation. Abundant clusters and scattered AFB in the subcutaneous and affected muscle.
17	15.34	3.38E+05	Yes	Ulcer	4.75E+05	Severe diffuse dermal and subcutaneous oedema with mixed inflammatory cell infiltrates, and necrosis. Severe multifocal necrotising vasculitis with AFB within the perivascular tissues, vascular wall and lumen. Skeletal muscle necrosis and oedema. Numerous clusters and scattered AFB in affected tissues.
17	16.17	2.50E+05	Yes	Ulcer	1.26E+04	Mild segmental epidermal hyperplasia, hyperkeratosis and moderate loss of epidermal appendages Severe diffuse dermal and subcutaneous oedema, inflammatory cell infiltrate and necrosis. Multifocal necrotising vasculitis. Multifocal skeletal muscle necrosis. Multifocal clusters and scattered AFB bacteria are present.
17	15.68	2.55E+05	Yes	Ulcer	2.36E+06	Moderate segmental epidermal hyperplasia and hyperkeratosis. Severe diffuse dermal and subcutaneous oedema, inflammatory cell infiltrate and necrosis. Severe multifocal necrotising vasculitis with acid-fast bacilli within the perivascular tissues, vascular wall, and lumen. Multifocal skeletal muscle necrosis. Multifocal clusters and scattered single AFB are present.
17	15.94	1.18E+05	Yes	Ulcer	3.05E+06	Mild to moderate subcutaneous oedema and multifocal tissue necrosis. Moderate chronic active inflammation. Scant clusters and scattered AFB.
19	16.32	1.61E+05	Yes	Ulcer	3.04E+06	Moderate diffuse dermal and subcutaneous oedema, multifocal mild vasculopathy, multifocal necrosis of skeletal muscle and connective tissues, mild mixed macrophage and lymphocyte infiltrations. Focal cluster of acid-fast bacilli close to necrotic tissue, scanty AFB in oedematous subcutaneous tissue.

20	15.95	8.36E+04	Yes	Ulcer	1.60E+06	Moderate dermal and subcutaneous oedema with multifocal necrosis of skeletal muscle fibres and interstitium with mixed inflammatory infiltrates of macrophages, lymphocytes and lesser numbers of neutrophils. There is segmental epidermal hyperplasia and hyperkeratosis. AFB are present in clusters with necrotic foci and scattered in low numbers within the oedematous tissues.

Table S5. Clinical and biometric features of C57BL/6 mice with lesions, and representative controls.

High dose

Mouse ID	Incubation (days)	Lesion onset to ulcer (days)	Change in weight (g)
8.1	64	13	1.0
8.2	60	N/A	N/A
8.3	60	6	-1.2
8.4	67	14	0.1
8.5	62	15	0.4
9.1	63	N/A	N/A
9.2	72	16	1.1
9.3	72	19	-0.2
9.4	65	18	1.5
9.5	63	N/A	N/A
10.1	64	13	-0.3
10.2	62	15	-0.9
10.3	53	13	3.9
10.4	67	10	0.8
10.5	67	15	0.3
Mean:	64	14	0.5*

Negative controls

9.1	N/A	N/A	0.4
9.2	N/A	N/A	0.1
9.3	N/A	N/A	-0.5
9.4	N/A	N/A	0.0
9.5	N/A	N/A	-0.2
10.1	N/A	N/A	0.4
10.2	N/A	N/A	0.3
10.3	N/A	N/A	0.3
10.4	N/A	N/A	0.8
10.5	N/A	N/A	0.0
Mean:	N/A	N/A	0.2*

*Student's t test, p = 0.382

Low dose

Mouse ID	Incubation (days)	Lesion onset to ulcer (days)	Change in weight (g)
19.1	77	18	1.5
19.2	81	24	1.7
19.3	77	25	5.6
19.4	72	23	-0.2
19.5	86	14	0.7
20.1	72	13	1.3
20.2	76	15	0.8
20.3	71	25	1.8
20.4	74	22	0.7
20.5	85	17	2.3
Mean:	77	20	1.6*

Negative controls

9.2	N/A	N/A	1.0
9.3	N/A	N/A	1.3
9.4	N/A	N/A	1.1
9.5	N/A	N/A	1.2
10.1	N/A	N/A	0.8
10.2	N/A	N/A	0.7
10.3	N/A	N/A	3.5
10.4	N/A	N/A	0.1
10.5	N/A	N/A	0.9
Mean:	N/A	N/A	1.2*

*Student's t test, p = 0.442

Table S6. Clinical and biometric features of BALB/c mice with lesions, and representative controls.

High dose

Mouse ID	Incubation (d)	Lesion onset to ulcer (days)	Change in weight (g)
4.1	85	24	1.0
4.2	91	27	1.0
4.3	82	20	-0.4
4.4	83	34	0.7
4.5	89	32	3.3
5.1	72	25	0.7
5.2	73	19	0.9
5.3	72	23	1.6
5.4	72	23	1.3
5.5	72	26	0.8
Mean:	79	25	1.1*

Negative controls

4.1	N/A	N/A	1.9
4.2	N/A	N/A	0.6
4.3	N/A	N/A	1.3
4.4	N/A	N/A	1.0
4.5	N/A	N/A	1.5
5.1	N/A	N/A	1.4
5.2	N/A	N/A	0.2
5.3	N/A	N/A	1.0
5.4	N/A	N/A	0.8
5.5	N/A	N/A	1.5
Mean:	N/A	N/A	1.1*

*Student's t test, p = 0.930

Low dose

Mouse ID	Incubation (d)	Lesion onset to ulcer (days)	Change in weight (g)
13.1	67	31	0.6
13.2	103	36	2.5
13.3	93	25	0.6
13.4	72	26	1.1
13.5	86	32	-0.1
14.1	81	37	0.2
14.2	105	29	1.5
14.3	73	25	0.9
14.4	79	38	1.8
14.5	79	35	0.5
Mean:	84	32	1.0*

Negative controls

5.1	N/A	N/A	0.3
5.2	N/A	N/A	0.5
5.3	N/A	N/A	1.7
5.4	N/A	N/A	1.3
5.5	N/A	N/A	1.6
Mean:	N/A	N/A	1.1*

*Student's t test, p = 0.773

Table S7. Differences between *M. ulcerans* dosing groups (high dose, low dose, and control) and various immune features over time (unadjusted univariate analysis).

C57BL/6

Parameter	Timepoint (weeks)	Location	Higher value	Lower value	p value
$CD8^+$ cells	4	Spleen	Low dose	Control	0.016
	4	Spleen	Low dose	High dose	0.016
	10	DLN	Low dose	Control	0.036
	> 10	DLN	Low dose	Control	0.021
$CD4^+$ cells	4	Spleen	Low dose	Control	0.016
	4	Spleen	Low dose	High dose	0.016
	> 10	DLN	Low dose	Control	0.017
T_{reg}	4	Spleen	Low dose	Control	0.016
	4	Spleen	Low dose	High dose	0.016
	10	Spleen	Control	Low dose	0.036
	> 10	DLN	Low dose	Control	0.008
B cells	4	Spleen	Low dose	Control	0.016
	4	Spleen	Low dose	High dose	0.016
	10	DLN	Low dose	Control	0.036
Plasma cells	4	Spleen	Low dose	Control	0.016
	4	Spleen	Low dose	High dose	0.032
	6	DLN	Low dose	Control	0.008
	>10	DLN	Low dose	Control	0.004
$\gamma\delta$ T cells	4	Spleen	Low dose	Control	0.016
	4	Spleen	Low dose	High dose	0.016
	6	DLN	High dose	Control	0.032
	> 10	DLN	Low dose	Control	0.002
	> 10	DLN	Low dose	High dose	0.029
NK cells	4	Spleen	Low dose	Control	0.016
	6	DLN	Low dose	Control	0.008
	6	DLN	High dose	Control	0.016
	> 10	DLN	Low dose	Control	0.017
	> 10	DLN	Low dose	High dose	0.019
Macrophages	4	Spleen	Low dose	Control	0.016
	4	Spleen	Low dose	High dose	0.016
	6	DLN	Low dose	Control	0.025
	6	DLN	High dose	Control	0.025
	10	DLN	Low dose	Control	0.036
	> 10	DLN	High dose	Control	0.036
	> 10	DLN	Low dose	Control	<0.001
	> 10	DLN	Low dose	High dose	<0.001
Neutrophils	4	Spleen	Low dose	High dose	0.016
	10	DLN	Low dose	Control	0.036
	> 10	Spleen	Low dose	Control	<0.001
Dendritic cells	4	Spleen	Low dose	Control	0.016
	4	Spleen	Low dose	High dose	0.032
	6	DLN	Low dose	Control	0.032
	> 10	DLN	Low dose	Control	0.003
	> 10	DLN	Low dose	High dose	0.012
IL-10	4	Serum	Control	High dose	0.012
CXCL10	6	Serum	Low dose	Control	0.012
CCL2 (MIP1)	6	Serum	Low dose	Control	0.034
	> 10	Serum	Low dose	Control	0.001
	> 10	Serum	High dose	Control	0.006
CXCL9	> 10	Serum	High dose	Control	0.045
VEGF	4	Serum	High dose	Control	0.045
	10	Serum	Low dose	Control	0.049
IL-4	6	Serum	Low dose	Control	0.016
IL-6	> 10	Serum	High dose	Control	0.002
	> 10	Serum	High dose	Low dose	0.005
IFN-α	> 10	Serum	High dose	Control	0.019

BALB/c

Parameter	Timepoint (weeks)	Location	Higher value	Lower value	p value
$CD8^+$ cells	10	Spleen	Control	Low dose	0.008
	10	Spleen	Control	High dose	0.032
$CD4^+$ cells	10	Spleen	Control	Low dose	0.016
	10	Spleen	Control	High dose	0.008
T_{reg}	10	Spleen	Control	Low dose	0.016
	10	Spleen	Control	High dose	0.008
B cells	6	DLN	Control	Low dose	0.032
	10	Spleen	Control	Low dose	0.008
	10	Spleen	Control	High dose	0.016
Plasma cells	4	DLN	Control	Low dose	0.032
	6	DLN	Control	Low dose	0.032
	10	Spleen	Control	Low dose	0.032
$\gamma\delta$ T cells	6	DLN	Control	Low dose	0.032
	10	Spleen	Control	Low dose	0.016
	10	Spleen	Control	High dose	0.032
NK cells	4	DLN	Control	High dose	0.032
	10	Spleen	Control	Low dose	0.016
	10	Spleen	Control	High dose	0.008
	10	DLN	Control	Low dose	0.008
	10	DLN	Control	High dose	0.008
Macrophages	4	DLN	Control	High dose	0.042
	10	Spleen	Control	Low dose	0.008
	10	Spleen	Control	High dose	0.008
Neutrophils	10	Spleen	Control	Low dose	0.016
Dendritic cells	10	Spleen	Control	Low dose	0.008
	10	Spleen	Control	High dose	0.008
IFN-α	4	Serum	Control	Low dose	0.016
IFN-γ	6	Serum	High dose	Low dose	0.045
CXCL9	4	Serum	High dose	Low dose	0.030
GM-CSF	6	Serum	Control	Low dose	0.025
IL-10	10	Serum	High dose	Control	0.025
	10	Serum	High dose	Low dose	0.044

Table S8. p values reaching statistical significance following correction for multiple analyses across the entire univariate dataset. C57BL/6 mice only are presented.

Parameter	Timepoint (weeks)	Location	Higher value	Lower value	p value	Adj. p value
$CD44^+$ $CD62L^{lo}$ $CD4^+$	> 10	DLN	Low dose	Control	0.002	0.038
$CD38^+$ $CD8^+$	> 10	DLN	Low dose	Control	0.003	0.038
$CD38^+$ $CD8^+$	> 10	DLN	Low dose	High dose	0.002	0.048

Table S9. p values reaching statistical significance following correction for multiple analyses across the dataset presented in Fig 5E. C57BL/6 mice only are presented.

Parameter	Location	p value	Adj. p value
$CD25^+$ $CD8^+$	Spleen	0.001	0.036
$KLRG1^+$ $CD8^+$	Spleen	0.010	0.036
GM-CSF	Serum	0.002	0.020
$CD107^+$ NK cells	DLN	0.013	0.050 (ns)

ns; not significant
